# Primary sex determination of placental mammals: a modelling study uncovers dynamical developmental constraints in the formation of Sertoli and granulosa cells

**DOI:** 10.1186/s12918-016-0282-3

**Published:** 2016-05-26

**Authors:** Lucas Sánchez, Claudine Chaouiya

**Affiliations:** Dpto. Biología Celular y Molecular, Centro de Investigaciones Biológicas (C. S. I. C.), c/Ramiro de Maeztu, 9, 28040 Madrid, Spain; Instituto Gulbenkian de Ciência – IGC, Rua da Quinta Grande, 6, P-2780-156 Oeiras, Portugal

**Keywords:** Placental mammals, Primary sex determination, Gene regulatory network, Logical modelling

## Abstract

**Background:**

Primary sex determination in placental mammals is a very well studied developmental process. Here, we aim to investigate the currently established scenario and to assess its adequacy to fully recover the observed phenotypes, in the wild type and perturbed situations. Computational modelling allows clarifying network dynamics, elucidating crucial temporal constrains as well as interplay between core regulatory modules.

**Results:**

Relying on a comprehensive revision of the literature, we define a logical model that integrates the current knowledge of the regulatory network controlling this developmental process. Our analysis indicates the necessity for some genes to operate at distinct functional thresholds and for specific developmental conditions to ensure the reproducibility of the sexual pathways followed by bi-potential gonads developing into either testes or ovaries. Our model thus allows studying the dynamics of wild type and mutant XX and XY gonads. Furthermore, the model analysis reveals that the gonad sexual fate results from the operation of two sub-networks associated respectively with an initiation and a maintenance phases. At the core of the process is the resolution of two connected feedback loops: the mutual inhibition of Sox9 and ß-catenin at the initiation phase, which in turn affects the mutual inhibition between Dmrt1 and Foxl2, at the maintenance phase. Three developmental signals related to the temporal activity of those sub-networks are required: a signal that determines Sry activation, marking the beginning of the initiation phase, and two further signals that define the transition from the initiation to the maintenance phases, by inhibiting the Wnt4 signalling pathway on the one hand, and by activating Foxl2 on the other hand.

**Conclusions:**

Our model reproduces a wide range of experimental data reported for the development of wild type and mutant gonads. It also provides a formal support to crucial aspects of the gonad sexual development and predicts gonadal phenotypes for mutations not tested yet.

**Electronic supplementary material:**

The online version of this article (doi:10.1186/s12918-016-0282-3) contains supplementary material, which is available to authorized users.

## Background

Sex determination in mammals results from two consecutive processes. The present work focuses on the *primary sex determination*, which refers to the development of the bi-potential, or indifferent, gonads along either the male (testis) or female (ovary) pathways. Once differentiated, the gonads direct the development of sexual dimorphic structures (*secondary sex determination*) that characterise the two sexes through the production of sex hormones [1, 2].

The bi-potential gonads are composed of somatic and germ cells. In mice, the somatic lineage arises at about 10.0 days post coitum (dpc) as a thickening of the coelomic epithelium on the mesonephros ventromedial surface. At this time, the gonads (or genital ridge) are identical in males and females (for detail on their formation, we refer to [3]). The primordial germ cells, germ line precursors (sperm and oocytes), originate outside the urogenital ridge where they are first detected at about 7.25 dpc; they then proliferate and migrate along the hindgut to the site of the forming gonad, which they populate between 10.0 and 11.0 dpc. The somatic cells of the bi-potential gonads are capable of adopting either the male or the female fate. The precursors of the “supporting somatic cells” (so named for their role in sustaining and nourishing germ cells, in both sexes) and the steroidogenic cells (which produce either male or female hormones) are present in the early gonads. The supporting cells develop into testis-specific Sertoli cells or into ovary-specific follicle (granulosa) cells. The differentiation of steroidogenic cells follows the specification of the supporting cell lineage. In testes, the anti-Müllerian hormone (Amh), secreted by the Sertoli cells, prevents the development of female genitalia and directs the differentiation of steroidogenic Leydig cells. These produce testosterone, inducing the development of male genitalia. In ovaries, the granulosa cells are involved in nourishing female germ cells and in converting androgens (secreted by steroidogenic theca cells) into oestrogens (reviewed in [4, 5]). In the present work, we focus on the differentiation of the supporting cells into testis specific Sertoli cells or ovary specific granulosa cells.

The key player for the sexual development of the bi-potential gonad is the Y-linked gene Sry (Sex-determining region Y), whose expression in XY gonads (from about 10.5 to 12.5 dpc, reaching is higher expression at about 11.5 dpc) determines testes development, whereas XX gonads develop into ovaries [6–8]. Sox9 (Sry-box 9), initially expressed in the bi-potential gonad of both sexes, is up-regulated by Sry in XY embryos, whereas it is down-regulated in XX embryos (at about 11.5 dpc) [9]. Sox9 up-regulation requires Sf1 protein (Steroidogenic factor 1) [10]. The gene Fgf9 (Fibroblast growth factor 9), initially expressed in the bi-potential gonads of both sexes, is up-regulated in XY gonads following the Sry-dependent increase of Sox9 expression [11]. Fgf9 participates in the inactivation of the (female) Wnt4 (Wingless related MMTV integration site 4) signalling pathway [11] and is required to maintain Sox9 high functional level [11–13]. The gene Dmrt1 (Doublesex- and mab-3-related transcription factor 1) is initially expressed at similar levels in male and female bi-potential gonads. It is later sex-specifically expressed in males, where it continuously represses the female sexual developmental programme [14–20].

The genes Wnt4 [11, 21–23] and Rspo1 (R-sponding 1) [24] are initially expressed in the bi-potential gonad of both sexes, but they are down regulated following Sry activation in XY while maintained in XX gonads. Both Wnt4 and Rspo1 have the same effector molecule, ß-catenin, indicating that they act together for ovarian development [25–29]. The gene Foxl2 (Forkhead-domain transcription factor L2), not expressed in the bi-potential gonad, is induced only in somatic cells of the ovary (at about 12.5 dpc) where it remains active [30–32]. It has been proposed that the Wnt4/Rspo1/ß-catenin signalling pathway controls the gonadal female differentiation of the gonad by repressing Sox9 during embryonic phases and that, later on Foxl2 takes over to ensure the ovarian identity maintenance [33].

The sexual development of the bi-potential gonad is determined during the narrow developmental time window that coincides with the time when Sry is expressed, so that if the testis pathway is not engaged at that time, the ovarian pathway ensues, becoming resistant to posterior Sry expression [34]. Thus, the correct timing of Sry expression is crucial in sex determination [35]. In addition, to induce testis development, Sry expression level must reach a certain threshold during this critical time window [36]. Another feature of the gonadal sexual development is that a critical number of differentiated Sertoli cells are required to ensure testis development [37, 38].

Available experimental data regarding primary sex determination is understood in the following terms [11, 39]. The developmental plasticity of the bi-potential gonad, caused by the antagonistic functions of the male Fgf9 and female Wnt4 signalling pathways, appears to be “programmed” to resolve in favour of Wnt4 pathway. However, the presence of Sry alters this resolution, favouring Fgf9 pathway, which determines testis development. This function of Sry is performed through its target Sox9, whose up-regulation leads to the increase of Fgf9 expression, which in turn inhibits Wnt4 pathway and assists Sox9 in maintaining its high expression level. Our goal is to investigate this established scenario and to determine its sufficiency to fully explain primary sex determination in placental mammals. To do so, we define a mathematical model of the gene regulatory network encompassing the major players identified so far. This modeling approach supports an integrative understanding of the inter-dependent behaviours of the genes involved. It further suggests the necessity of additional players to ensure a correct functioning of the mechanisms at stake.

Focusing on the mechanisms controlling the fate determination of a common cell population towards either Sertoli or granulosa cells, Rios et al. recently defined a Boolean model of a regulatory network encompassing a set of genes well known for their involvement in primary sex determination in mammals [40]. About 30 % of the interactions of this network were inferred relying on the model dynamical analysis to match expected behaviours. This previous work shows that, despite the likely involvement of a greater number of players, a core regulatory network seems enough to drive this complex developmental process. The authors further point to the requirement of ß-catenin for the female development and the putative role ß-catenin in regulating Foxl2. Here, due to the scarcity of quantitative data, we also relied on a logical modelling approach. In contrast, while all the interactions of our model were supported by experimental data, we predicted the requirement of temporal signals to drive the dynamics of the core network. Furthermore, we resorted to an extended modelling formalism supporting the consideration of multi-valued variables, which allowed to further dissect the roles ß-catenin. Besides, by properly connecting two instances of the regulatory network, we could elucidate the observed central-to-polar asymmetry in the differentiation of Sertoli cells.

## Methods

The model was defined using the logical formalism [41, 42] and the software tool GINsim [43]. Further details are provided in the Additional file 1. Briefly, the gene regulatory network is represented as a directed graph, whose nodes and arcs stand for the genes and their interactions, respectively. Each node is assigned a discrete variable that describes the node state, with a maximal level defining the highest qualitative functional level of the regulatory node (this maximal level equals 1 in the simplest, Boolean case). Whenever distinct functional concentrations of a regulatory product need to be considered, multilevel variables are used. Each arc embodies a regulatory interaction and is assigned a threshold, which defines the smallest functional level of the interaction source for which the interaction is operative. Logical parameters qualitatively describe the effects of the regulatory interactions controlling the states of the network nodes. The definition of the model dynamics according to a given updating scheme (synchronous, asynchronous or specific priorities), as well as simulations of mutant conditions are described in the Additional file 1.

## Results

**Fig. 1:**
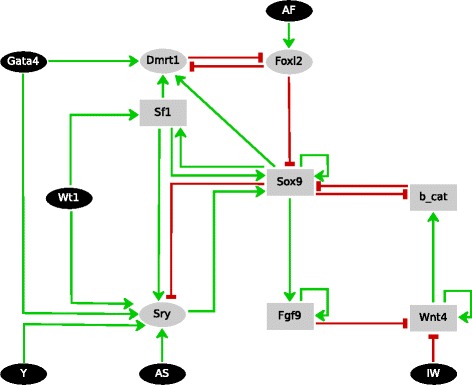
The (simplified) gene regulatory network controlling primary sex determination in placental mammals (see Additional file 1: Figure S1 for the more complete network). Ellipsoids and rectangles represent Boolean and multi-valued variables, respectively. Black nodes correspond to inputs. Normal green and blunt red arrows represent positive and negative interactions, respectively. Y stands for Y chromosome, which contains the gene Sry; AS stands for the developmental signal that allows Sry activation; AF stands for the developmental signal that activates Foxl2; and IW stands for the developmental signal that inhibits the Wnt4 signalling pathway; b-cat stands for ß-catenin. Description of these temporal signals is provided in the Additional file

We first assembled the regulatory network with the genes known to be involved in the primary sex determination of placental mammals (Additional file 1: Figure S1). Then, for simplicity’s sake, we performed a set of reductions that do not affect the basic biological features of the regulatory network, obtaining the sub-network shown in Fig. 1. Additional file 1 explains this reduction process and reviews the experimental results backing each interaction of the gene network of Fig. 1. Next, the inputs Gata4, AS (activator of Sry), IW (Inhibitor of Wnt4 pathway) and AF (activator of Foxl2) were defined, assuming that they account for “developmental temporal signals” acting on the gene network. Their functions are described in the Additional file 1.

For parsimony, we first assumed that the genes (and their products) have a single functional threshold value (represented by Boolean variables). However, the behaviour of the resulting model could not reproduce the biological process under study, indicating that some components ought to have additional functional levels to make the model simulate the process. This was the case for Sf1, Sox9, Fgf9, Wnt4 and ß-catenin, all associated with two distinct functional levels (the corresponding variables can take three values: 0, 1 and 2). The justification for these multilevel variables is given in the Additional file and values ranges and logical parameters of the model components are provided in Additional file 1: Table S2. First, stable states of the model were identified, since these embody potential differentiated cellular states. Contrary to the 3 stable states produced by the Rios et al.’s model that likely result from the interactions added to fit the model [40], our model gives rise to a large number of stable states [27] including states accounting for the testis and ovary phenotypes. This number is greatly reduced when considering the relevant combinations of input values (i.e., male/female and initiation/maintenance external signals, as described below) and even more when selecting relevant initial conditions (see Additional file 1). As a conclusion, the present model indicates that input signals and starting state matter for selecting the appropriate differentiation pathway of the gonad.

To analyse the dynamics of the gene network, the final sexual state (testis or ovary) adopted by the gonad was considered to result from two processes: the *initiation phase* that refers to the transition (entrance) of the gonad from its un-differentiated state to its sexual pathway, and the *maintenance phase* that culminates in the final state. From the formal point of view,

1. The *initiation phase* was defined by the functions of Gata4 on Sry and Dmrt1 expression and of the developmental signal AS on Sry expression.
2. The *maintenance phase* was defined by the functions of the developmental signals IW on Wnt4 pathway and AF on Foxl2 expression, together with the lack of AS and Gata4 signals on Sry, and of Gata4 on Dmrt1 expression.

**Fig. 2:**
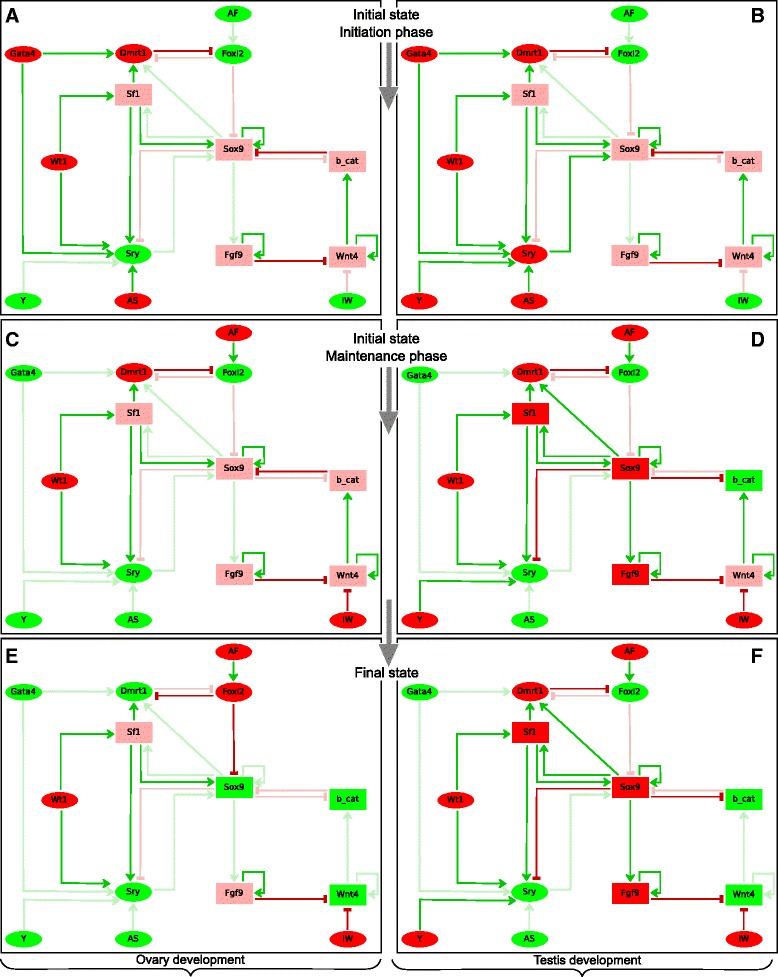
Dynamics of the gene regulatory network. The two phases of the primary sex determination process, initiation (**a**–**b**) and maintenance (**c**–**d**), are represented for the XX and XY bi-potential gonads. The final state is represented in **e**–**f**. The green, pink and red colours respectively represent null, intermediate and highest level of the corresponding gene. The strong and faded colours of the arrows indicate operative and non-operative interactions, respectively. For the remaining symbols, see legend of Fig. 1

From a modelling point of view, the initiation and maintenance phases would correspond to the operation of two sub-networks of the sex determination network (Fig. 2). To construct the dynamics, the final state of the initiation sub-network was taken as the initial state of the maintenance sub-network, for which AS and Gata4 were switched off and AF and IW were switched on.

The model asynchronous dynamics of the wild type XX gonad and the Fgf9 KO XY gonad includes key bifurcation points, whose resolution determines irreversibly the gonadal fate. Qualitative restrictions regarding the rates at which specific genes change their functional levels were therefore expressed in terms of priorities (see Additional file 1: Figure S2). It is worth noting that the deterministic synchronous behaviour does not allow such predictions. Indeed, we could verify that, under a synchronous update, the model simulations lead to the expected dynamics as illustrated in the Fig. 2.

The model simulation (using a synchronous update or asynchronous priority classes) thus recapitulated the development of the wild type bi-potential gonad into either ovary or testis (Fig. 2). Since XX and XY gonads are identical, the simulations started from initial states differing only in the status of Y (present or not).

1. *Development of the XX bi-potential gonad (*Fig. 2*, left)*. The state vector does not change along the initiation phase, entering the maintenance phase when AF activates Foxl2, which in turn represses Dmrt1 (whose function cannot be maintained because Sox9 is also repressed), and when IW inhibits Wnt4/ß-catenin. The final state (ovary) is reached and maintained by Foxl2 function.

2. *Development of the XY bi-potential gonad (*Fig. 2*, right)*. The initiation phase starts when the developmental signal AS allows Sry activation, which in turn raises Sox9 expression from its initial level 1 to 2. As a consequence, Sf1 and Fgf9 expression levels increase and ß-catenin becomes inhibited, allowing the maintenance of Sox9 high functional level. Moreover, Sox9 and Fgf9 high levels lead to Sry repression and Wnt4-signalling pathway inhibition. Importantly, Sox9 high functional level maintains Dmrt1 expression along the transition from the initiation to the maintenance phases: when the developmental signals AF and IW are triggered and AS and Gata4 signals fade away, AF cannot activate Foxl2 because of Dmrt1 presence. This determines the final state reached by the gonad (testis) maintained by the continuous expression of Dmrt1.

**Fig. 3:**
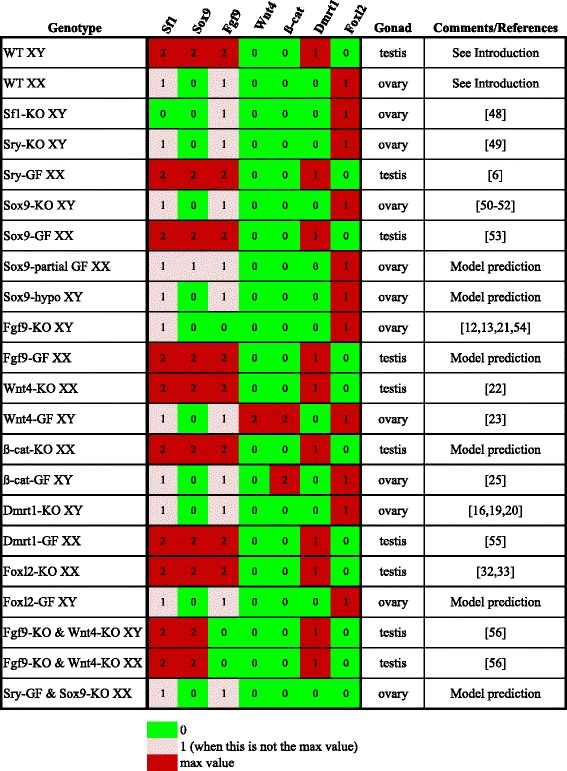
Final stable states reached by the gene network and the corresponding phenotypes (testis, ovary) for the gonad under wild type and mutant conditions. The left column indicates the genotype of the gonad; the middle seven columns provide the gene levels; and the right column shows the sexual phenotype developed by the gonad. “KO” stands for knock-out (loss-of-function), “GF” stands for gain-of-function, “hypo” stands for partial loss-of-function. The colour code is described in legend of Fig. 1

A series of perturbations of the sex determination regulatory network were simulated in the form of single and double loss-of-function mutations, as well as ectopic expression experiments. Here again, we verified that the resulting differentiated states were obtained for both the synchronous update and the asynchronous priorities. To define the sexual phenotypes of the final states resulting from model simulations, we used the following criteria: expression of Sox9 and Dmrt1 and absence of Foxl2 indicate a testicular identity, while Foxl2 expression and absence of both Sox9 and Dmrt1 denote an ovarian identity (details in the Additional file 1). The results, summarised in Fig. 3, all agreed with experimental observations when available [44–56] or provided a set of predictions:

1. Sox9 partial loss-of-function XY gonad develops into ovary, whereas Sox9 partial gain-of-function XX gonad still gives rise to ovary.
2. XX gonad carrying gain-of-function Fgf9 mutations leads to a testis phenotype.
3. Gain-of-function Foxl2 mutation determines ovarian development of XY gonads.
4. XX gonad double mutant for Sry gain-of-function and Sox9 loss-of-function results into ovary.

The sexual phenotypes of the gonads carrying either loss-of-function (KO) or gain-of-function (GF) mutations in the genes encoding the developmental temporal signals AS, Gata4, IW and AF were simulated. Results, detailed in the Additional file 1, predicted the following phenotypes:

1. AS-KO XY and AS-GF XX gonads develop into ovaries.
2. Gata4-KO XY gonad develops into ovary, whereas Gata4-GF XX gonad leads to a testis phenotype.
3. IW-KO XX gonad develops into ovary, while IW-GF XY and IW-GF XX are predicted to develop into testes.
4. AF-KO XX and AF-GF XY gonads develop into testes.

Finally, we simulated additional model perturbations, by suppressing the auto-regulations of Sox9, Wnt4 and Fgf9. In the case of Sox9, while having no effect in a XX gonad, this alteration leads to an ovary phenotype of the XY gonad. For Wnt4, the suppression of the auto-regulation affects the development of the XX gonad, which adopts a testis phenotype (no effect for the XY gonad). The suppression of Fgf9 auto-regulation maintains the multi-stability observed in the asynchronous dynamics of the Fgf9-KO (Additional file 1: Figure S2), but when considering a synchronous update or the proposed priorities, the XY gonad adopts an ovary phenotype. Altogether, these results showed the requirement of these interactions, which are indeed documented in the literature (see Additional file 1).

**Fig. 4:**
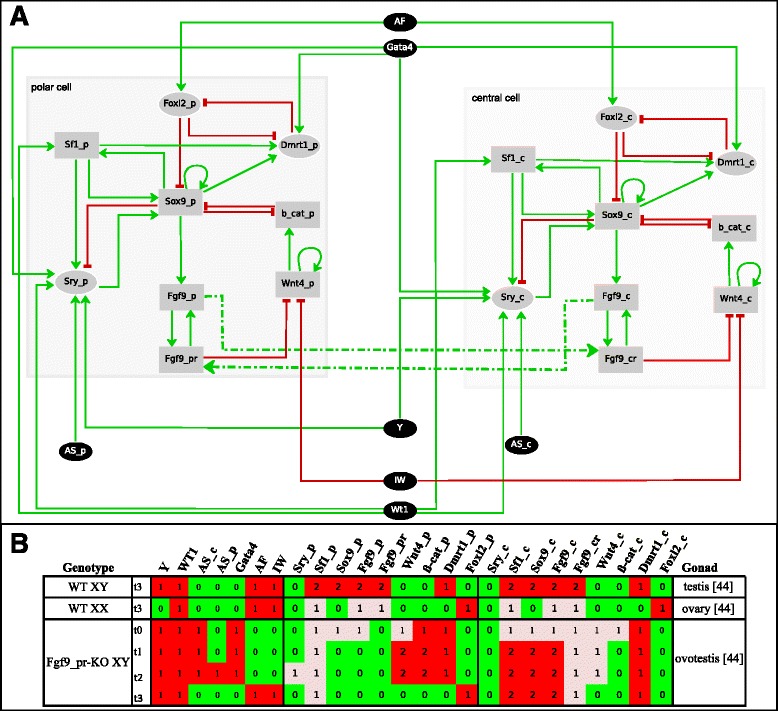
Spatio-temporal induction pattern of Sertoli cells during the development of the bi-potential XY gonad into testis. **a** The 2-cell network represents the central (“c”) and pole (“p”) regions of the gonad; the suffixes “cr” and “pr” indicate the Fgf9 receptor of the central and pole cells, respectively. The dashed lines denote Fgf9 paracrine function. t0 stands for the bi-potential gonad initial state; t1 stands for the activation of Sry in the central region of the gonad; t2 stands for the activation of Sry in the pole regions of the gonad, and t3 stands for the maintenance state. For details see text and Additional file 1. **b** Final states reached by the 2-cell network and the corresponding phenotypes (testis, ovary and ovotestes) for the gonad under wild type and mutant conditions for the paracrine function of Fgf9. Remaining symbols and colour codes are given in the legend of Fig. 1

As mentioned in the Background, to develop into testis, the bi-potential gonad needs the induction of a threshold number of Sertoli cells. This induction first occurs in the centre and afterwards in the gonadal poles, paralleling Sry temporal activation. Moreover, induction of Sertoli cells in the poles requires the Fgf9 signal from the centre towards the poles so that Fgf9 failure produces ovotestes with male tissue in the poles and ovarian tissue in the central region [53]. To model this process, two replicas of the 1-cell network of Fig. 1 were connected, defining a new 2-cell network (Fig. 4a and Additional file 1). The phenotypes resulting from this 2-cell model are shown in Fig. 4b. The simulated results regarding the wild type XY and XX gonads, as well as the failure of Fgf9 signalling from the central to the polar region of an XY gonad agreed with experimental results. Moreover, model analyses suggested that the cells at the pole region would not need the later activation of Sry to become Sertoli cells. This would be due to a putative Fgf9-relay mechanism originating from the centre and spreading towards the poles, provided this mechanism operated within the narrow time window of the gonadal sexual determination. Thus, the central-to-pole asymmetry in the differentiation pattern of Sertoli cells would be a consequence of the earlier activation of Sry in the central region. Reverting the normal situation formally proved this: *in silico* experiment where Sry was first activated in the pole region and later in the centre showed the formation of ovotestes with male tissue in the pole and ovarian tissue in the centre (data not shown). Additionally, this result provides an explanation for the rare cases where the ovotestes are formed by ovarian tissue in the gonadal centre and testis tissue in the poles [54]: these ovotestes would result from any perturbation causing Sry activation in the poles earlier than in the centre. It has been reported that the Wnt4 signalling pathway does not play a role in the spatiotemporal induction of Sertoli cells in XY gonads by analysing heterozygous Wnt4 (+/−) XY gonads [53]. Model simulation of homozygous Wnt4 (−/−) formally supports that contention (data not shown).

XY and XX gonads simultaneously mutant for Sox9 and ß-catenin express both testis and ovary genes, with XY showing more masculinisation than XX gonads. This seems to be caused by the function of Sox8 that partially surmounts lack of Sox9 [55]. Our model provides a formal explanation for this observation (see Additional file 1: Figure S1 for details). In XY mutant gonad, Sry expression, which persists longer than in wild type gonads because of the absence of its repressor Sox9, together with the absence of ß-catenin could lead to Sox8 activation causing Dmrt1 expression.

Finally, all the genetic perturbations analysed for the 1-cell network reproduced the same phenotypes in the case of the 2-cell network (data not shown).

## Discussion

Gathering the current knowledge of the gene network controlling primary sex determination in placental mammals in the form of a computational, logical model, we could determine specific constraints to get this network behave in accordance with bi-potential gonad sexual differentiation. More precisely, the model dynamical analysis, for wild type and mutant XX and XY gonads, showed that:

1. The sexual development of the bi-potential gonad cannot be explained with a Boolean model: at least two distinct functional levels are required to convey the roles of Sf1, Sox9, Fgf9, Wnt4 and ß-catenin.
2. The final sexual state reached by the gonad results from two processes, initiation and maintenance, each associated with the operation of a sub-network of the gene regulatory network.
3. Three developmental signals, related to the temporal sexual pattern of the gonad, are required. The timing of Sry activation is defined by an activator signal (AS). Following the initiation phase, when the maintenance phase begins, a signal inhibits Wnt4 pathway (IW), and another signal activates Foxl2 (AF). These signals operate independently of the sexual fate of the bi-potential gonad and their molecular nature remains to be established.
4. Sox9 auto-regulation operates already at the bi-potential state of the gonad, though it cannot be stably set up, i.e., it cannot bring Sox9 to its highest functional level because ß-catenin activity prevents it.
5. The previously proposed antagonistic function (balance) of male Fgf9 and female Wnt4 receives a formal demonstration. Additionally, this balance is found to be implemented by a core module composed by the Sox9 and ß-catenin exclusive feedback loop whose resolution determines the sexual fate adopted by the gonad.
6. The mutual repression of Dmrt1 and Foxl2 underlies the maintenance of the adopted sexual fate, either testis or ovary. The positive interaction of Sox9 upon Dmrt1 is required for the continuous function of Dmrt1 when Gata4, its initial non-sex specific activator, is no longer present.
7. The primary role of Sry is to boost Sox9 expression to overcome inhibition from ß-catenin. This determines the persistence of Dmrt1 function to prevent the non-sex specific developmentally programmed activation of Foxl2.
8. Qualitative restrictions regarding the rates at which specific genes change their functional levels were identified: level increases of Wnt4, ß-catenin, Foxl2 and level decreases of Dmrt1, Fgf9r should be faster than any changes in the levels of the remaining network components. The requirement of a faster increase of Wnt4 functional level is operationally related to the faster decrease of its inhibitor, the Fgf9 receptor (Fgf9r). Similarly, the requirement of a faster increase of ß-catenin (effector molecule of the Wnt4 signalling pathway) is operationally related to the faster increase of its activator, Wnt4. These conditions serve the same biological process: prevention of the establishment of Sox9 high expression during the initiation phase thanks to the inhibition that ß-catenin exerts on Sox9. Finally, the requirement of a faster decrease of Dmrt1 functional level is related to the faster increase of Foxl2, since these two genes repress each other. This last condition serves the same biological process, establishment and maintenance of one of the alternative final states, testis or ovary.

The temporal transcriptome analysis of the gonad shows that both male- and female-determining genes are expressed, with an over-representation of the latter ones [56]. This led to suggest that the female programme might constitute the “default” state: in the absence of additional inputs, the bi-potential gonad would follow the female pathway by inhibiting the expression of male-promoting genes [56]. The theoretical analysis presented here supports this proposal and leads to the following summary description of the process.

In the XX bi-potential gonad, Sf1 activates Sox9 and both ß-catenin and Sox9, engaged in a mutual inhibitory loop, are maintained at low functional levels. As time goes by, the on-going function of Wnt4-signalling pathway supplies functional ß-catenin so that Sox9 expression starts to decay. Accordingly, the function of Fgf9 signalling pathway decreases, reinforcing the function of Wnt4 signalling pathway so that ß-catenin continues to be supplied into the system. When the gonad reaches the developmental time when Foxl2 is activated, this activation is made possible because Dmrt1 expression cannot be maintained —following lack of Gata4 function and low Sox9 expression. The end result is that Foxl2 activation leads to a final repression of Sox9 and Dmrt1, ensuring and maintaining the ovarian identity of the gonad.

Recall that the gonadal fate is “determined” during a narrow developmental time window, which coincides with the expression time of Sry in wild type XY gonads. When Sry is activated, its product boosts Sox9 expression to its higher level, overcoming the inhibitory effect of ß-catenin. The higher functional level of Sox9 increases Fgf9 signalling pathway, whereas Wnt4 signalling and then ß-catenin become inhibited; consequently, high expression of Sox9 is maintained. At the time in development when Foxl2 becomes activated, this activation is prevented by Dmrt1, which is maintained—after the lack of Gata4 function—by the high expression of Sox9. Consequently, Dmrt1 drives and maintains the testis identity of the gonad.

## Conclusion

The construction and analysis of our logical model indicated that the final sexual fate of a bi-potential gonad would result from the temporal action of two sub-networks respectively associated with an initiation and a maintenance phase. Moreover, this fate would ensue from successive resolutions of two connected feedback loops: the mutual repression of Sox9 and ß-catenin at the initiation phase, which in turn would affect the resolution of the mutual repression of Dmrt1 and Foxl2 at the maintenance phase. Three developmental signals related to the activity of the two sub-networks would be required: a signal determining the time of Sry activation that marks the initiation phase onset, and two further signals that define the transition from the initiation to the maintenance phases, by inhibiting the Wnt4 signalling pathway on the one hand, and by activating Foxl2 on the other hand.

The relevance of our model is demonstrated through the reproduction of a wide range of experimental data reported for the development of wild type and mutant gonads. It further provides a formal support to crucial aspects of the gonad sexual development and predicts gonadal phenotypes for mutations that have not been yet tested experimentally.

## Additional file

Additional file 1: (1) *Logical modelling framework:* basics, simulation of genetic perturbations, updating schemes and Hierarchical Transition Graphs. (2) *Gene network controlling primary sex determination in placental mammals:* Simplification of the regulatory network, experimental results backing the interactions of the gene network of Fig. 1, definition of developmental temporal signals acting on the gene network. (3) *Logical model definition and analysis:* genes having more than a single functional level, logical parameters, stable state analysis, temporal constraints and definition of priorities. (4) *Model analyses for mutant gonads:* mutations of the sex determination genes, mutations of the developmental temporal signals, genetic redundancy in primary sex determination. (5) *Threshold number of Sertoli cells required for gonadal development into testis: the 2-cell network*. (6) **Figure S1.** The male and female genes that have been identified and their proposed interactions involved in the sexual development of the gonad in placental mammals. (7) **Figure S2.** Hierarchical transition graphs revealing bifurcations in the discrete dynamics and the required restrictions on the delays to ensure the reachability of the expected final stable state. (8) **Figure S3.** Final states reached by the gene network and their corresponding phenotypes (testis, ovary) for the gonad under mutant conditions (loss**-**of-function and gain**-**of-function) of the developmental signals. (9) **Table S1.** official names of the components of the network displayed in Additional file 1: Figure S1. (10) **Table S2.** Maximal values and logical parameters defining the effect of regulatory interactions, for each model component. (PDF 821 kb)

## References

[1] Brennan J, Capel B (2004). One tissue, two fates: molecular genetic events that underlie testis versus ovary development. Nat Genet.

[2] Munger SC, Capel C (2012). Sex and the circuitry: progress toward a systems-level understanding of vertebrate sex determination. WIREs Syst Biol Med.

[3] Eggers S, Sinclair A (2012). Mammalian sex determination—insights from humans and mice. Chromosome Res.

[4] Ewen KA, Koopman P (2010). Mouse germ cell development: from specification to sex determination. Mol Cell Endocrinol.

[5] Wainwright EN, Wilhelm D (2010). The game plan: celular and molecular mechanisms of mammalian testis development. Curr Top Dev Biol.

[6] Koopman P, Gubbay J, Vivian N, Goodfellow P, Lovell-Badge R (1991). Male development of chromosomally female mice transgenic for *Sry*. Nature.

[7] Kashimada K, Koopman P (2010). Sry: the master switch in mammalian sex determination. Development.

[8] Larney C, Bailey TL, Koopman P (2014). Switching on sex: transcriptional regulation of the testis-determining gene Sry. Development.

[9] Sekido R, Bar I, Narváez V, Penny G, Lovell-Badge R (2004). SOX9 is up-regulated by the transient expression of SRY specifically in Sertoli cell precursors. Dev Biol.

[10] Sekido R, Lovell-Badge R (2008). Sex determination involves synergistic action of SRY and SF1 on a specific Sox9 enhancer. Nature.

[11] Kim Y, Kobayashi A, Sekido R, DiNapoli L, Brennan J, Chaboissier MC (2006). Fgf9 and Wnt4 act as antagonistic signals to regulate mammalian sex determination. PLoS Biol.

[12] Kim Y, Bingham N, Sekido R, Parker KL, Lovell-Badge R, Capel B (2007). Fibroblast growth factor receptor 2 regulates proliferation and Sertoli differentiation during male sex determination. Proc Natl Acad Sci U S A.

[13] Bagheri-Fam S, Sim H, Bernard P, Jayakody I, Taketo MM, Scherer G (2008). Loss of Fgfr2 leads to partial XY sex reversal. Dev Biol.

[14] Raymond CS, Kettlewell JR, Hirsch B, Bardwell VJ, Zarkower D (1999). Expression of Dmrt1 in the genital ridge of mouse and chicken embryos suggests a role in vertebrate sexual development. Dev Biol.

[15] De Grandi A, Calvari V, Bertini V, Bulfone A, Peverali G, Camerino G (2000). The expression pattern of a mouse doublesex-related gene is consistent with a role in gonadal differentiation. Mech Dev.

[16] Raymond CS, Murphy MW, O’Sullivan MG, Bardwell VJ, Zarkower D (2000). *Dmrt1*, a gene related to worm and fly sexual regulators, is required for mammalian testis differentiation. Genes Dev.

[17] Lei N, Hornbaker KI, Rice DA, Karpova T, Agbor VA, Heckert LL (2007). Sex-Specific Differences in Mouse DMRT1 Expression Are Both Cell Type- and Stage-Dependent During Gonad Development. Biol Reprod.

[18] Fahrioglu Y, Murphy MW, Zarkower D, Bardwell VJ (2007). mRNA Expression Analysis and the Molecular Basis of Neonatal Testis Defects in *Dmrt1* Mutant Mice. Sex Dev.

[19] Kim S, Bardwell VJ, Zarkower D (2007). Cell type-autonomous and non-autonomous requirements for Dmrt1 in postnatal testis differentiation. Dev Biol.

[20] Matson CK, Murphy MW, Sarver AL, Griswold MD, Bardwell VJ, Zarkower D (2011). DMRT1 prevents female reprogramming in the postnatal mammalian testis. Nature.

[21] Schmahl J, Kim Y, Colvin JS, Ornitz DM, Capel B (2004). Fgf9 induces proliferation and nuclear localization of FGFR2 in Sertoli precursors during male sex determination. Development.

[22] Vainio S, Heikkila M, Kispert A, Chin N, McMahon AP (1999). Female development in mammals is regulated by Wnt-4 signalling. Nature.

[23] Jordan BK, Shen JHC, Olaso R, Ingraham HA, Vilain E (2003). Wnt4 overexpression disrupts normal testicular vasculature and inhibits testosterone synthesis by repressing steroidogenic factor 1/ß-catenin synergy. Proc Natl Acad Sci U S A.

[24] Parma P, Radi O, Vidal V, Chaboissier MC, Dellambra E, Valentini SL (2006). R-spondin1 is essential in sex determination, skin differentiation and malignancy. Nat Genet.

[25] Maatouk DM, DiNapoli L, Alvers A, Parker KL, Taketo MT, Capel B (2008). Stabilization of ß-catenin in XY gonads causes male-to-female sex-reversal. Hum Mol Genet.

[26] Tevosian SG, Manuylov NL (2008). To ß or not to ß: canonical ß-catenin signaling pathway and ovarian development. Dev Dyn.

[27] Kim KA, Zhao J, Andarmani S, Kakitani M, Oshima T, Binnerts ME (2006). R-spondin proteins: a novel link to ß-catenin activation. Cell Cycle.

[28] Chassot AA, Ranc F, Gregoire EP, Roepers-Gajadien HL, Taketo MM, Camerino G (2008). Activation of *β*-catenin signaling by Rspo1 controls differentiation of the mammalian ovary. Hum Mol Genet.

[29] Tomizuka K, Horikoshi K, Kitada R, Sugawara Y, Iba Y, Kojima A (2008). R-spondin1 plays an essential role in ovarian development through positively regulating Wnt-4 signaling. Hum Mol Genet.

[30] Schmidt D, Ovitt CE, Anlag K, Fehsenfeld S, Gredsted L, Treier AC (2004). The murine winged-helix transcription factor Foxl2 is required for granulosa cell differentiation and ovary maintenance. Development.

[31] Ottolenghi C (2007). Loss of Wnt4 and Foxl2 leads to female-to-male sex reversal extending to germ cells. Hum Mol Genet.

[32] Ottolenghi C, Pelosi E, Tran J, Colombino M, Douglass E, Nedorezov T (2005). Foxl2 is required for commitment to ovary differentiation. Hum Mol Genet.

[33] Uhlenhaut NH, Jakob S, Anlag K, Eisenberger T, Sekido R, Kress J (2009). Somatic sex reprogramming of adult ovaries to testes by FOXL2 ablation. Cell.

[34] Hiramatsu R, Matoba S, Kanai-Azuma M, Tsunekawa N, Katoh-Fukui Y, Kurohmaru M (2009). A critical time window of *Sry* action in gonadal sex determination in mice. Development.

[35] Bullejos M, Koopman P (2005). Delayed Sry and Sox9 expression in developing mouse gonads underlies B6-Y^DOM^ sex reversal. Dev Biol.

[36] Nagamine CM, Morohashi K, Carlisle C, Chang DK (1999). Sex reversal caused by *Mus musculus domesticus* Y chromosomes linked to variant expression of the testis-determining gene *Sry*. Dev Biol.

[37] Palmer SJ, Burgoyne PS (1991). In situ analysis of fetal, prepuberal and adult XX–XY chimaeric mouse testes: Sertoli cells are predominantly, but not exclusively, XY. Development.

[38] Schmahl J, Capel B (2003). Cell proliferation is necessary for the determination of male fate in the gonad. Dev Biol.

[39] Kim Y, Capel B (2006). Balancing the Bipotential Gonad Between Alternative Organ Fates: A New Perspective on an Old Problem. Dev Dyn.

[40] Ríos O, Frias S, Rodríguez A, Kofman S, Merchant H, Torres L (2015). A Boolean network model of human gonadal sex determination. Theor Biol Med Model.

[41] Thomas R, D’Ari R (1990). Biological feedback.

[42] Chaouiya C, Remy E, Mossé B, Thieffry D (2003). Qualitative analysis of regulatory graphs: a computational tool based on a discrete formal framework. Lecture notes in control and information science.

[43] Chaouiya C, Naldi A, Thieffry D (2012). Logical modelling of gene regulatory networks with GINsim. Methods Mol Biol, Bact Mol Netw (Part 3).

[44] Luo X, Ikeda Y, Parker KL (1994). A cell-specific nuclear receptor is essential for adrenal and gonadal development and sexual differentiation. Cell.

[45] Gubbay J, Vivian N, Economou A, Jackson D, Goodfellow P, Lovell-Badge R (1992). Inverted repeat structure of the Sry locus in mice. Proc Natl Acad Sci U S A.

[46] Chaboissier MC, Kobayash A, Vidal VIP, Lützkendorf S, van de Kant HJG, Wegner M (2004). Functional analysis of Sox8 and Sox9 during sex determination in the mouse. Development.

[47] Barrionuevo F, Bagheri-Fam S, Klattig J, Kist R, Taketo MM, Englert C (2006). Homozygous Inactivation of Sox9 Causes Complete XY Sex Reversal in Mice. Biol Reprod.

[48] Lavery R, Lardenois A, Ranc-Jianmotamedi F, Pauper E, Gregoire EP, Vigier C (2011). XY Sox9 embryonic loss-of-function mouse mutants show complete sex reversal and produce partially fertile XY oocytes. Dev Biol.

[49] Vidal VPI, Chaboissier MC, de Rooij DG, Schedl A (2001). Sox9 induces testis development in XX transgenic mice. Nat Genet.

[50] Colvin JS, Green RP, Schmah J, Capel B, Ornitz DM (2001). Male-to-Female Sex Reversal in Mice Lacking Fibroblast Growth Factor 9. Cell.

[51] Lindeman RE, Gearhart MD, Minkina A, Krentz AD, Bardwell VJ, Zarkower D (2015). Sexual cell-fate reprogramming in the ovary by DMRT1. Curr Biol.

[52] Jameson SA, Lin YT, Capel B (2012). Testis development requires the repression of Wnt4 by Fgf signaling. Dev Biol.

[53] Hiramatsu R, Harikae K, Tsunekawa N, Kurohmaru M, Matsuon I, Kanai Y (2010). FGF signaling directs a center-to- pole expansion of tubulogenesis in mouse testis differentiation. Development.

[54] Eicher EM, Beamer WG, Washburn LL, Whitten WK (1980). A cytogenetic investigation of inherited true hermaphroditism in BALB/cWt mice. Cytogenet Cell Genet.

[55] Nicol B, Yao HHC (2015). Gonadal Identity in the Absence of pro-Testis Factor SOX9 and pro-Ovary Factor beta-catenin in mice. Biol Reprod.

[56] Jameson SA, Natarajan A, Cool J, DeFalco T, Maatouk DM, Mork L (2012). Temporal Transcriptional Profiling of Somatic and Germ Cells Reveals Biased Lineage Priming of Sexual Fate in the Fetal Mouse Gonad. PLoS Genet.

