# Supplementary material for: Primary sex determination of placental mammals: a modelling study uncovers dynamical developmental constraints in the formation of Sertoli and granulosa cells
Source: BMC Syst Biol. 2016 May 26;10:37. doi: 10.1186/s12918-016-0282-3 (PMC4880855; doi:10.1186/s12918-016-0282-3)
Supplement: Additional file 1: — (1) Logical modelling framework: basics, simulation of genetic perturbations, updating schemes and Hierarchical Transition Graphs. (2) Gene network controlling primary sex determination in placental mammals: Simplification of the regulatory network, experimental results backing the interactions of the gene network of Fig. 1, definition of developmental temporal signals acting on the gene network. (3) Logical model definition and analysis: genes having more than a single functional level, logical parameters, stable state analysis, temporal constraints and definition of priorities. (4) Model analyses for mutant gonads: mutations of the sex determination genes, mutations of the developmental temporal signals, genetic redundancy in primary sex determination. (5) Threshold number of Sertoli cells required for gonadal development into testis: the 2-cell network. (6) Figure S1. The male and female genes that have been identified and their proposed interactions involved in the sexual development of the gonad in placental mammals. (7) Figure S2. Hierarchical transition graphs revealing bifurcations in the discrete dynamics and the required restrictions on the delays to ensure the reachability of the expected final stable state. (8) Figure S3. Final states reached by the gene network and their corresponding phenotypes (testis, ovary) for the gonad under mutant conditions (loss-of-function and gain-of-function) of the developmental signals. (9) Table S1. official names of the components of the network displayed in Additional file 1: Figure S1. (10) Table S2. Maximal values and logical parameters defining the effect of regulatory interactions, for each model component. (PDF 821 kb) [file 12918_2016_282_MOESM1_ESM.pdf]

# Supporting text


**1. LOGICAL MODELLING FRAMEWORK** 1

*1.1. Basics* 2

**1.2. Simulation of genetic perturbations** 3

*1.3. Updating schemes and Hierarchical Transition Graphs* 3

**2. GENE NETWORK CONTROLLING PRIMARY SEX DETERMINATION IN PLACENTAL MAMMALS** 5

**2.1. Simplification of the regulatory network** 5

**2.2. Experimental results backing the interactions of the gene network of Fig. 1** 7

**2.3. Definition of developmental temporal signals acting on the gene network** 10

**3. LOGICAL MODEL DEFINITION** 11

**3.1. Genes having more than one single functional level** 12

**3.2. Logical parameters** 13

**3.3. Stable states analysis** 13

**3.4. Temporal constraints; defining priorities** 13

**4. MODEL ANALYSES FOR MUTANT GONADS** 14

**4.1. Mutations of the sex determination genes** 14

**4.2. Mutations of the developmental temporal signals** 17

**4.3. Genetic redundancy in primary sex determination** 18

**5. THRESHOLD NUMBER OF SERTOLI CELLS REQUIRED FOR GONADAL DEVELOPMENT INTO TESTIS: THE 2-CELL NETWORK.** 20

**6. REFERENCES** 21


# 1. Logical modelling framework

## *1.1. Basics*

The model presented in this work was defined using the logical formalism (1, 2). In this framework, the gene regulatory network is represented in terms of a directed graph. Each node (gene or regulatory product) is assigned a discrete variable with a maximal level, which defines the highest qualitative functional level taken by the regulatory node (this maximal level equals 1 in the simplest, Boolean case). Whenever distinct functional concentrations of a regulatory product need to be considered, multilevel variables are used. Each arc embodies a regulatory interaction and is assigned a threshold. This threshold defines the smallest functional level of the interaction source for which the interaction is operative. Logical parameters qualitatively describe the effects of each interaction or combination of interactions controlling the states of the network nodes. Thus, each node is associated to as many parameters as the number of combinations of interactions acting on this node. Per default, these parameters are set to zero. Defining non zero parameters amounts to single out the combinations of interactions enabling the activation of the targeted node and to qualitatively recover all documented wild type and mutant gene expression patterns. Parameters for the 1-cell network of Fig. 1 are given in the Supplementary Table S2.

A state of the network is represented by a vector, which encompasses the current (discrete) node levels. Given a state, one can determine which interactions are operative and the values of the logical parameters indicate the nodes called to change their levels (i.e. those nodes whose current levels differ from the values of the logical parameters). In general, for a given state, all possible elementary transitions (i.e. switching of a single node level, to a neighbouring integer level) are considered, thus leading to as many outgoing transitions as updating calls (asynchronous updating, see Section 1.3). Depending on the structure of the regulatory graph and on the values of the logical

parameters, a model leads to a large but finite number of dynamical pathways. These are in turn represented in the form of a **State Transition Graph (**STG), where nodes represent states and (directed) edges represent transitions between states.

The software tool GINsim (3) was used for the simulation and logical analysis of the gene regulatory network. The model files for the 1-cell and 2-cell networks are available as a Supplementary Files F1 and F2, in SBML format (4). They will be also provided in the model repository of the GINsim software web site (http://ginsim.org), where the tool is freely available.

## 1.2. Simulation of genetic perturbations

In the logical framework, simulation of genetic perturbations is straightforward. A loss-of-function mutation of a given gene implies that this gene produces a non-functional product (or no product at all), which amounts to assign the value zero to the corresponding variable and parameters. In contrast, the ectopic expression of a gene implies that this gene is expressed in an unregulated manner beyond its normal spatio-temporal expression domain. This can be accomplished by forcing the corresponding variable to take higher values (for detail of this formal treatment of mutations, see (5).

## *1.3. Updating schemes and Hierarchical Transition Graphs*

Model dynamics are defined by the specification of initial state(s) and of an updating scheme, which refers to how network component levels (i.e., model variables) are updated. In a synchronous update scheme, at each simulation step, all the components that are called to change their levels are simultaneously updated. This defines a deterministic behaviour in which each state has at most one successor. In contrast, the asynchronous update scheme is considered to be more realistic since it accounts for different activation and/or inactivation time of the model components. This update

amounts to generate all possible trajectories from the specified initial conditions: e.g. when two component levels are changed, these updates are done asynchronously, defining two transitions towards alternative successor states. The resulting STG may then include bifurcation states from which, depending on the choice of the successor state, the final stable state may differ; in other words, in the absence of any further constraint, two alternative phenotypes are reachable.

For our model of the gene network controlling primary sex determination, constraints on conflicting updates (transitions) needed to be considered to get the final state match the expected phenotype (testis or ovary). Such constraints are expressed in terms of priorities, i.e. temporal orders between component updates (6). To uncover these priorities, we relied on a compact representation of the dynamics, called **Hierarchical transition graphs** (HTG) (Supplementary Fig. S2).

HTG have been introduced as compact representations revealing the attractors and their basins of attraction, as well as transient oscillatory behaviours (7). To briefly describe the compaction leading to these graphs, we first recall that a **Strongly Connected Component** (SCC) is defined as a maximal strongly connected subgraph (i.e., a maximal subset of states in the STG, such that there is a path connecting each state to any other state). These SCCs are termed *trivial* when they encompass a single state, *complex* otherwise.

States of an STG are gathered into single nodes of the corresponding HTG as follows:

- If they belong to a complex SCC;
- If they belong to an attractor i.e. a trivial or a complex SCCs with no outgoing transitions;
- If they define trivial SCCs from which the same complex and terminal SCCs are

reachable.

Hence nodes of an HTG are defined as sets of states. The arcs between these nodes denote the existence of (at least) a transition between two states of the corresponding sets of states. They can be labelled by the associated updates of variables of the logical model (Supporting Fig. 2). Importantly, a path in a HTG towards any node defined by a complex or terminal SCC implies a path in the original STG (7).

# 2. Gene network controlling primary sex determination in placental mammals

In this section, the components and the interactions included in the gene network are discussed.

## 2.1. Simplification of the regulatory network

For simplicity matters, before defining the logical model, we performed a series of simplification of the detailed gene network (Supplementary Fig. S1), which resulted in the network illustrated in Fig. 1. These simplifications are described and justified in what follows.

The sexual cyto-differentiation genes, such as Amh and Fst were removed since they constitute the output, male versus female, of the regulatory network. Pgd2 was also eliminated because it is not essential for testis development (8). Gonad development into ovary requires the function of the canonical Wnt4 signalling pathway. This is triggered by both Wnt4 and Rspo1, which operate through the same effector, ß-catenin. For simplification, we selected the secreted ligand Wnt4 to represent Wnt4 signalling pathway.

The gene Sox9 plays a key, crucial role for testis determination, yet its function seems to

be dispensable for maintaining adult testis identity (9). It appears that this function is exerted by its homolog Sox8, which is activated by Sox9 after its Sry-dependent up-regulation (10). This is so because the elimination of Sox8 function in XY mice does not affect the development of the testis (11), except that they are unfertile, suggesting that Sox8 is required for the maintenance of male fertility in the adult (12). On the other hand, Dmrt1 binds to the Sox9 and Sox8 promoters and in Dmrt1(-/-) XY mutant gonads their expression was reduced (13). The interactions between Sox9, Sox8 and Dmrt1 shown in Fig. S1 were simplified and instead of considering both Sox9 and Sox8 in the genetic network, it was only considered Sox9 and assumed that participates in persistent testis identity through its interaction with Dmrt1 (see below).

The receptors Fgfr2 and Fzd/Lrp of the secreted ligands Fgf9 and Wnt4, respectively, were eliminated since, from a formal point of view, their functional states follow that of their ligands; i.e., receptors are merely intermediate components between ligand and effector molecules of the corresponding signalling pathways. Consequently, the positive interactions from Fgfr2 to Fgf9 and from ß-catenin to Wnt4 represent the autocrine functions of Fgf9 and of Wnt4, respectively. They were replaced by positive self-loops on Fgf9 and on Wnt4. The paracrine function of Fgf9 is represented by a negative interaction towards Wnt4. For simplicity, we assumed that Foxl2 is capable of exerting its function without the support of its co-factor, the oestrogen receptor ERα. Finally, we considered that, unless the presence of Dmrt1 prevents it, Foxl2 is constitutively activated by 12.5 dpc in both sexes due to a developmentally programmed activator (see Section 2.3).

The positive interaction of Dmrt1 upon Sox9 was eliminated because it was found to be irrelevant for gonadal sex determination.

## 2.2. Experimental results backing the interactions of the gene network of Fig. 1

***Wt1-Sf1 interaction.*** Wt1 is required to initiate Sf1 expression because, even at the earliest time when Sf1 expression can be observed at 9.5 dpc (14), Sf1 expression is absent in both XY and XX embryos mutant for Wt1 (15). Moreover, Wt1 binds to a 674 bp fragment in the Sf1 promoter causing its activation (15).

***Wt1-Sry and Gata4-Sry interactions***. As previously mentioned, several factors have been identified that, when mutated, prevent the initiation of the male program because Sry expression is very much affected (reviewed in (16) and cites therein). Wt1 and Gata4 were chosen to represent these activators of Sry.

***Sf1-Sry interaction.*** *In vitro* transfection studies showed that Sf1 protein binds to and activates Sry promoter (17, 18). Both XY and XX mice mutant for Sf1 develop female genitalia (19). Since the absence of either Sf1 or Wt1 causes male-to-female sex reversal, proper activation of Sry requires the presence of both Sf1 and Wt1.

***Sry-Sox9 and Sf1-Sox9 interactions.*** Sox9 is a direct target of Sry (20, 21). Chromatin immune-precipitation assays revealed that Sry protein binds to the TESCO enhancer sequence in the Sox9 promoter (22). Up-regulation of Sox9 requires both Sry and Sf1 activators: Sf1 can activate TESCO *in vitro* although at low levels by itself, and Sry is able to activate TESCO *in vitro* only in the presence of Sf1 (22). Moreover, ubiquitous expression of Sry in XX gonads results in the initiation of Sox9 expression only in Sf1-positive cells (23).

***Sox9-Sox9 interaction.*** Sox9 can bind the TESCO element of its own promoter via the same Sry binding sites (22). However, by itself, Sox9 has no activity in co-transfection assays, requiring the presence of Sf1 to activate TESCO (22). Indeed, a direct interaction

between the N-terminal domain of Sox9 and the C-terminal domain of Sf1 has been described for the regulation of Amh promoter (24).

***Sox9-Sf1 interaction.*** *In vitro* transfection analysis indicated that Sox9 could activate Sf1 promoter in a reporter construct with increasing amounts of Sox9 (25).

***Sox9-Sry interaction.*** A negative feedback turns off Sry transcription, but only if the level of Sry protein is above a critical threshold required to initiate testis development (20). This is consistent with findings where Sry expression was prolonged in sex-reversed ovotestes in embryos carrying a weak allele of Sry (26). This negative feedback is mediated by Sox9 since the rapid decrease of Sry expression occurs just after Sox9 activation: Sry expression is not turned off at 12.5-13.5 dpc in XY gonads with low levels of Sox9 (10) or in XY gonads where Sox9 function is inactivated (27).

***Sox9-Fgf9 interaction.*** Fgf9 expression decreases significantly or is absent in Sox9(-/-) XY gonads at 11.5 dpc, suggesting that Fgf9 expression in wild-type XY gonads depends on Sox9 expression (28).

***Fgf9-Wnt4 interaction.*** At 12.5 dpc, Wnt4 is up-regulated in Fgf9(-/-) XY gonads but not in wild type XY gonads. This suggests that Fgf9 is necessary for the down-regulation of Wnt4 in differentiating XY gonads (28). Moreover, treatment of XX gonads with exogenous Fgf9 suppresses Wnt4 normal expression (28).

***Indirect activation of Sox9 by Fgf9, through Wnt4.*** In XY gonads mutant for Fgf9, the initial and up-regulation steps of Sox9 are not affected, but Sox9 is lost later on, indicating that Fgf9 is required to maintain and not initiate Sox9 function (29). On the other hand, it was shown that the deletion of Wnt4 function prevents the male-to-female

sex transformation of XY Fgf9 mutants, indicating that Fgf9 indirectly activates Sox9 through the inhibition of Wnt4 function (30).

***Wnt4-ß-catenin interaction.*** ß-catenin is the effector of the Wnt4-signalling pathway (reviewed in (31).

***Sox9-ß-catenin and ß-catenin-Sox9 interactions.*** Co-immune-precipitation experiments showed that Sox9 and ß-catenin form a physical complex that results in their mutual destruction by ubiquitination/26S proteasome pathway. The C-terminal transactivation domain of Sox9 was needed for its inhibitory activity on ß-catenin. *In vitro* binding assays showed that deletion mutants of Sox9 that lack the C-terminal transactivation domain fail to interact with ß-catenin (32).

***Foxl2-Sox9 interaction.*** Homozygous null mutations for Foxl2 result in Sox9 up-regulation in XX ovaries after birth (33). The transcription factor Foxl2 represses TESCO activity *in vitro* and chromatin immune-precipitation assays demonstrated that Foxl2 is indeed bound to the TESCO sequence. Moreover, inactivation of all identified binding sites for Foxl2 in the TESCO element results in a de-repression of TESCO activity in the adult ovary *in vivo* (34).

***Foxl2-Dmrt1 interaction.*** Four Foxl2-binding sequences have been identified in Dmrt1 promoter. Increasing the amount of co-transfected Foxl2 causes a decrease in Dmrt1 transcription and removal of those putative Foxl2-like binding sequences prevents this Foxl2-dependent repression (35).

***Dmrt1-Foxl2 interaction.*** Dmrt1-knockout XY mice are born as males, although their testes later develop abnormally, so that Dmrt1 is crucial to maintain the testicular function (36). In conditional knockout mice, where Dmrt1 is specifically ablated in foetal

Sertoli cells or even in the testes of adult males, Foxl2 expression is induced. This strongly indicates that Dmrt1 prevents Foxl2 expression (13).

***Sox9-Dmrt1 interaction.*** Here, we assumed that the maintenance of Dmrt1 testis-expression depends on Sox9 as XY gonads lacking Sox9 function show a reduced Dmrt1 expression (37). It could be argued that this may be explained by Foxl2 activation in those gonads (27, 37), causing Dmrt1 repression (see above). This would imply a direct repression of Foxl2 by Sox9, which has not been reported. Indeed, experimental evidence argues against such a direct interaction of Sox9 on Foxl2: in Sox9(-/-) XY gonads, Foxl2 starts to be activated at its normal time (i.e., 12.5 dpc, (38) as in wild type XX gonads (27). Alternatively, Sox9 negative effect on Foxl2 could be indirect, through Dmrt1: Sox9 would be required for Dmrt1 expression that, in turn, would repress Foxl2. In the absence of Sox9 function, Dmrt1 expression could not be maintained and consequently Foxl2 activation would follow. This interpretation agrees with the temporal appearance of Sox9 and Dmrt1 in ovarian cells upon Foxl2 deletion: Sox9 expression precedes that of Dmrt1, suggesting that Sox9 transcriptionally regulates Dmrt1 (34, 39). Interactions defining Sox9, Dmrt1 and Foxl2 relationships in Fig. 1 were based on this interpretation.

## 2.3. Definition of developmental temporal signals acting on the gene network

***Gata4-Dmrt1 interaction.*** It is known that Gata4 is an activator of Dmrt1 (40-42). However, Gata4 requirement to regulate Dmrt1 expression is not constant: Gata4 is obligatory to initiate Dmrt1 expression in the bi-potential gonad of both sexes and to maintain Dmrt1 expression in Sertoli cells in early phases of testis development, but subsequent persistence of Dmrt1 expression does not require Gata4.

***AS-Sry interaction.*** Although Sry activators, here represented by Wt1 and Gata4, are

already expressed in the bi-potential gonad, Sry is not activated. This activation occurs later, suggesting a temporal control of expression of Sry. We hypothesised the existence of AS (Activator of Sry) to account for the time in development when Sry expression appears.

***IW-Wnt4 interaction.*** An inhibitor of Wnt4 pathway (IW) was hypothesised to account for the subsequent lack of effect of the Wnt4 pathway on gonadal sexual development.

***AF-Foxl2 interaction.*** How Foxl2 would be activated under the above scenario? Since Dmrt1 represses Foxl2, it could be that the initial, non-sex specific, Dmrt1 expression prevents Foxl2 activation before Sry activation, key event in male development. However, this is not the case as Dmrt1(-/-) XY mutant gonads develop into testes and become feminised after birth (36, 43). Thus, Dmrt1 does not seem to participate in male development triggering, but rather appears to maintain testis identity in adult males (37). Similarly, the alternative that Sox9 could repress Foxl2 does not seem to be correct because, as mentioned above, Foxl2 activation starts at its normal time in XY gonads mutant for Sox9 as it is in wild type XX gonads (27). Here, through the introduction of a putative Activator of Foxl2 (AF), we proposed that Foxl2 activation in both XY and XX gonads is constitutive and developmentally programmed by 12.5 dpc. Hence, in XY gonads, this Foxl2 activation would be overcome by Dmrt1 expression, which represses Foxl2. However, in XX gonads, as Dmrt1 is not expressed at that time, Foxl2 would be activated.

# 3. Logical model definition

Having defined the regulatory components and interactions to be included in the network, we are ready to define the logical model, i.e. specify the ranges taken by the variables associated to the components, their logical parameters, and finally the updating

scheme to be considered for the model dynamics (see Section 1). This section justifies the requirement for multi-valued variables for some components, lists the logical parameters of our 1-cell network model and finally, discusses the temporal constraints found to ensure a final phenotype (i.e. in each condition (XX vs XY gonad), the reachability of the expected final state,).

## 3.1. Genes having more than one single functional level

***Sf1 functional levels.*** In the bi-potential gonad of both sexes, the gene Sf1 is initially activated by Wt1 and, following Sry activation, becomes up-regulated in the XY but not in the XX gonad (25). We thus assumed that Sf1 initial expression corresponds to its first functional level, whereas its Sox9-dependent up-regulation corresponds to its second functional level. This second functional level is justified because Sox9 function requires Sf1 as co-factor and because Sox9 up-regulation produces more Sox9 protein, requiring a higher amount of Sf1.

***Sox9 functional levels.*** It was found that Sox9 auto-regulation needed to be operative already at its initial expression level in the bi-potential gonad for the model reproduce its sexual development. Hence, we assumed that Sox9 initial activation by Sf1 corresponds to its first functional level, and that subsequent expression of Sry brings Sox9 to its second functional level. This second level would be required to trigger the male development of the bi-potential gonad.

***Fgf9 and Wnt4 functional levels.*** We assumed that the initial expression of both Fgf9 and Wnt4 (level 1) relates to their roles in cell proliferation during the formation and developmental plasticity of the bi-potential gonad. The specific up-regulation of Fgf9 in XY gonads after the increase of Sox9 expression (28) would constitute Fgf9 second functional level, involved in the inactivation of Wnt4-signalling pathway (28). Wnt4

second level would reflect its up-regulation in XX gonads, being involved in the inhibition of Sox9 auto-regulation (28).

***ß-catenin functional levels.*** Being the effector of the Wnt4-signalling pathway (reviewed in (31), ß-catenin levels reflect those of Wnt4.

## 3.2. Logical parameters

The values of the logical parameters define the behaviour of the regulatory network (see Section 1.1). These parameters (listed in the Supplementary Table S2) were progressively specified by comparing simulation results with published experimental data, describing wild type and mutant phenotypes, and by applying simplicity criteria; that is, the lowest parameter value was selected whenever different values were compatible with available data.

## 3.3. Stable states analysis

GINsim enables the identification of all the stable states of a logical model. Using this feature, 27 stable patterns were identified (data not shown). However, this number greatly decreases if we restrict the input values to relevant combinations (see Supporting Table S3). These results suggest that the temporal signals are instrumental for the selection of the adopted differentiated state. Morever, as shown by the model simulations, the selection of a relevant initial condition leads to trajectories that recapitulate observed differentiation decisions by reaching the Sertoli or granulosa cell phenotypes.

## 3.4.Temporal constraints; defining priorities

Analysing the model dynamics (Supplementary Fig. S2), we could define priorities on well-chosen transitions ensuring the sole reachability of the expected stable state: faster transitions (priority class 1) were those increasing the levels of Wnt4, ß-catenin

and Foxl2 and those decreasing the levels of Dmrt1, whereas all remaining transitions were considered slower. Note that transitions increasing Wnt4 and ß-catenin occur at the initiation phase, whereas Foxl2 increase and Dmrt1 decrease occur at the maintenance phase.

# 4. Model analyses for mutant gonads

## 4.1. Mutations of the sex determination genes

A series of perturbations of the sex determination logical model were simulated in the form of single and double loss-of-function mutations, as well as ectopic expression experiments (see Section 1.2). To define the sexual phenotypes of the final states obtained when simulating the model, we used the following criteria: expression of Sox9 and Dmrt1 and absence of Foxl2 indicate a testicular identity, while Foxl2 expression and absence of both Sox9 and Dmrt1 denote an ovarian identity. The results, discussed below, are summarised in Fig. 3.

**1.** Loss-of-function of Sf1 caused ovarian development for both XX and XY gonads, in agreement with experimental results (44).

**2.** XY gonad lacking Sry function developed into ovary (45), whereas gain-of-function Sry mutation in XX gonad developed into testis, in agreement with experimental observations (46).

**3.** XY gonad lacking Sox9 function developed into ovary (10, 27, 37) and XX gonad constitutively expressing Sox9 gave rise to testis, in agreement with experimental results (47). Moreover, model simulations predicted that Sox9 partial loss-of-function XY gonad would develop into ovary, whereas Sox9 partial gain-of-function XX gonad still gave rise to ovary.

**4.** Fgf9 loss-of-function caused XY gonad to develop into ovary, in agreement with experimental observations (28,29,48,49). Moreover, XX gonad carrying gain-of-function Fgf9 mutations was predicted to develop into testis. This result deserves further comments. To our knowledge, no experiments on the development of the bi-potential gonad constitutively expressing Fgf9 have been performed. However, wild type XX bi-potential gonads have been exposed to exogenous Fgf9 —by culturing the gonads into a solution containing Fgf9. Two contradictory results have been reported: on the one side, the gonads did not express testis markers such as Sox9 (49), whereas on the other side, Sox9 expression (testis development) was reported, suggesting that Sox9 activation by exogenous Fgf9 is caused by Wnt4 function inhibition (28). This discrepancy is ascribed to the use of intact gonads in the first case and of dissociated XX gonads in the second case; i.e., it is explained by a lower accessibility of Fgf9 to the cells in the negative case of Sox9 activation (28). To assess this scheme, model simulations were performed accounting for these *in vitro* experiments, by considering dissociated (Fgf9 accessibility) or intact (Fgf9 non-accessibility) gonads exposed to exogenous Fgf9. To this end, the initial state was that of the wild type XX gonad except that Fgf9=2, for the dissociated gonad (Fgf9=1 for the intact gonad); i.e., the high or low accessibility of Fgf9 was reflected by the high or low concentration of Fgf9 in the cell at the initial state. In the case of a dissociated gonad, the exogenous Fgf9 induced Sox9 expression, whereas such induction did not occur for an intact gonad (data not shown). These results provided a formal demonstration to the explanation of (28).

**5.** Lack of Wnt4 function in XX gonad resulted in testis development (50), whereas Wnt4 ectopic expression in XY gonad resulted in ovary development, in agreement with experimental results (51). As previously mentioned, ß-catenin is the effector molecule of Wnt4 signalling pathway. Accordingly, mutations in ß-catenin are expected to mimic the

mutations in Wnt4. This was indeed the case as XX gonad lacking ß-catenin function was predicted to develop as testis. Furthermore, XY gonad constitutively expressing ß-catenin developed as ovary, in agreement with experimental observations (52).

**6.** It has been reported that XY gonads lacking Dmrt1 function initially develop as normal testes but become feminised later (36, 43). The same effect has been reported when Dmrt1 function is removed from foetal or adult testes (13). The simulation of XY gonad lacking Dmrt1 function agreed with those experimental observations, in accordance with the role played by Dmrt1 in maintaining testis identity. In addition, gain-of-function Dmrt1 mutations were predicted to determine testis development of XX gonad. In this respect, it has been reported very recently that the expression of a conditional Dmrt1 transgene in the ovary silences the female sex-maintenance gene Foxl2 and consequently re-programmes juvenile and adult female granulosa cells into male Sertoli-like cells (53).

**7.** It has been reported that XX gonads lacking Foxl2 function initially develop as normal ovaries but become masculinised later (33). Moreover, the loss of Foxl2 function in adult ovaries causes their trans-differentiation into testes (34). Simulating XX gonad lacking Foxl2 function reproduced those experimental observations, in accordance with the role of Foxl2 in maintaining ovarian. Moreover, gain-of-function Foxl2 mutations were predicted to determine the ovarian development of XY gonads.

**8.** Simulation of both XY and XX gonads carrying simultaneously loss-of-function Fgf9 and Wnt4 mutations develop as testes, in agreement with experimental results (30).

**9.** XX gonad double mutant for Sry gain-of-function together with Sox9 loss-of-function developed into ovary. To our knowledge, such a combination has not been experimentally tested yet. This result formally demonstrated that Sry action on male gonadal development takes place through the Sox9 gene.

## 4.2. Mutations of the developmental temporal signals

The sexual phenotypes of the gonads carrying either loss-of-function (KO) or gain-of-function (GF) mutations in the genes encoding the developmental temporal signals AS, Gata4, IW and AF were simulated. The predicted phenotypes follow.

**1.** AS-KO XY gonads are predicted to develop into ovaries similarly to Sry-KO XY gonads, since Sry activation does not occur. However, AS-GF XX gonads would develop into ovaries instead of testes as in the case of Sry-GF XX gonads. This is so because XX gonads do not carry the Sry gene.

**2.** Gata4-KO XY gonads are expected to develop into ovaries similarly to Sry-KO XY gonads, since Sry activation does not occur. However, Gata4-GF XX gonads would develop into testes similarly to Sry-GF or Dmrt1-GF XX gonads. This is so because the persistent expression of Gata4 maintains Dmrt1 expression, thus preventing Foxl2 activation.

**3.** IW-KO XX gonads are expected to develop into ovaries instead of testes as in the case of Wnt4-KO XX gonads. This is so because the lack of IW function allows the continuous function of the Wnt4-signalling pathway. This prevents Sox9 to reach its highest functional level and consequently the expression of Dmrt1 cannot be maintained. Hence, Foxl2 becomes activated and ovary development ensues. IW-GF XY gonads develop into testes in contrast to Wnt4-GF XY gonads that develop into ovaries. This is so because in IW-GF XY gonads, Sox9 reaches its highest functional level so that Dmrt1 maintains its function, thus preventing Folx2 activation. IW-GF XX gonads are predicted to develop into testes. This is so, because IW inhibits the Wnt4-signalling pathway, leading to a resolution of the Sox9-ß-catenin feedback loop in favour of Sox9 —due to the lack of ß-catenin activity— so that Sox9 auto-regulatory function get this gene to

reach its highest functional level. This, in turn, maintains the expression of Dmrt1, thus preventing Foxl2 activation.

**4.** AF-KO XX gonads are predicted to develop into testes similarly to Foxl2-KO XX gonads. However, AF-GF XY gonads are expected to develop into testes instead of ovaries as in the case of Foxl2-GF XY gonads, because the initial expression of Dmrt1 prevents Foxl2 activation by AF.

## 4.3. Genetic redundancy in primary sex determination

Nicol and Yao´s study of the expression of sexual dimorphic genes during early sexual determination of XY and XX gonads simultaneously mutant for Sox9 and ß-catenin, demonstrates genetic redundancy (54). These authors proposed three classes of gonads: ovary class (expressing female genes), testis class (expressing male genes) and intersex class (expressing both male and female genes). The ovary class includes the wild type XX and Sox9-KO XY gonads. The wild type XY gonads form the testis class. The intersex class is defined by the ß-catenin-KO XX gonads together with the XX and XY gonads double mutant for Sox9-KO and ß-catenin-KO (hereafter double-KO gonads). Interestingly, the intersexual double-KO XY gonads clearly show more masculinisation than the double-KO XX gonads. The authors argued that this difference is due to a subset of male genes being up-regulated only in the double-KO XY gonads. Among these genes, some are Sry-targets such as, for example, Sox8. In addition, Nicol and Yao observed was that Sry expression lasts longer in double-KO than in wild type XY gonads. Lavery et al. (55) reported similar results for XX and XY gonads double mutants for Sox9 and Rspo1 genes.

Our model accounts for these results (see complete network of Fig. S1). To this respect, the ensuing observations are relevant for explanation purposes. First Sox8 shows a

significant degree of homology to Sox9 at the DNA-binding HMG box and its two flanking regions and a lower homology at the C-terminal region (56). Secondly, Sox8 is turn on in the male gonad by Sox9 after this is up-regulated in a Sry-dependent manner (57, 58). Although Sox8 is a direct target of Sry (59), its main activator is Sox9 as the transcription of Sox8 directly depends on Sox9 expression (58). Thirdly, Sox8 activates the gene Amh, although this activation is weaker compared to Sox9-dependent activation (57), likely due to the differences between Sox8 and Sox9 in the C-terminal transactivation domain. And fourth, Sox9, throughout its C-terminal domain, interacts with ß-catenin resulting in their mutual destruction (32). These observations led us to assume that Sox8 and ß-catenin do interact also to form a complex that results in their mutual destruction, that is, that Sox8 and ß-catenin are engaged in a feedback loop.

Following our model, the higher degree of masculinisation of double-KO XY intersexual gonads compared to double-KO XX gonads is due to the continuing Sry expression — caused by the absence of its repressor Sox9— and to the absence of ß-catenin so that Sox8 can be activated, allowing the maintenance of Dmrt1 expression in the double-KO XY gonads with the consequent masculinisation effect. This agrees the reported experimental results regarding the complementary expression pattern of Dmrt1 and Foxl2 in double-KO XX *vs* XY gonads: in the XX mutants, the great majority of cells express Foxl2 whereas the few remaining cells express Dmrt1; in XY mutants, however, there is a significant increase in the numbers of cells expressing Dmrt1 and the remaining cells express Foxl2 (54).

The finding that double-KO XY gonads do not show a full male-to-female transformation as in the case of single Sox9-KO XY gonads can also be explained in the light of our model. In the latter mutant gonads —carrying wild type ß-catenin—, the initial Sox9-ß-catenin feedback loop is resolved in favour of ß-catenin since there is no Sox9 function.

Consequently, ß-catenin expression level rises, thus preventing Sox8 activation despite of the persistent Sry function. Hence, Foxl2 activation occurs since Dmrt1 cannot maintain its expression in the absence of both Sox9 and Sox8. Notice that the effect of Sry on Sox8 activation is irrelevant in wild type XY gonads because the Sox9-ß-catenin feedback loop is resolved in favour of Sox9, which represses Sry.

# 5. Threshold number of Sertoli cells required for gonadal development into testis: the 2-cell network.

To address this question, two replicas of the 1-cell network of Fig. 1 were connected, defining a new network (Fig. 4A): the replica identified as network "c" represents the gonadal centre and the replica identified as network "p" represents the gonadal pole. In this 2-cell network, the autocrine and paracrine functions of Fgf9 were explicitly dissociated: two positive arrows between Fgf9 and its receptor (Fgf9r) account for Fgf9 autocrine function, whereas the positive arrow from Fgf9 of one network towards Fgf9-receptor of the other network accounts for Fgf9 paracrine function. For the 2-cell model, the values of the logical parameters were those of the 1-cell model, with the corresponding modifications for the components involved in the new interactions (see Supporting Table S2).

In addition, to get the 2-cell network dynamics mimics the biological process, it was necessary to refine the temporal constraints previously defined, as a consequence of the inclusion of Fgf9-receptor (Fgf9r). Thus, faster transitions (priority class 1) were those increasing the levels of Wnt4, ß-catenin and Foxl2 and those decreasing the levels of Dmrt1, Fgf9r (in both cells), whereas all remaining transitions were considered slower.

The distinct temporal activation of Sry in central *versus* pole regions was modelled by assuming that AS, the developmental activator of Sry, exerts its function earlier in the

central region —represented by AS_c— than in the pole region —represented by AS_p. Hence, the initiation phase was subdivided into two sub-phases (t1 and t2), and the input signals were defined by: at t1, presence of Y, Wt1, Gata4, AS_c and absence of AS_p, AF and IW, and at t2, presence of Y, Wt1, Gata4, AS_c, AS_p and absence of AF and IW. In this scenario, the final state of t1 was taken as initial state of t2, whose final state was the initial state of the maintenance phase (t3).

# 6. References

1. Thomas R, D'Ari R. *Biological Feedback*. CRC Press**,** Boca Raton, Florida, USA. 1990.

2. Chaouiya C, Remy E, Mossé B, Thieffry D. Qualitative analysis of regulatory graphs: a computational tool based on a discrete formal framework. *Lecture Notes in Control and Information Science*. Springer Berlin Heidelberg. 2003; 294:119–126.

3. Chaouiya C, Naldi A, Thieffry D. Logical Modelling of Gene Regulatory Networks with GINsim, *Methods in Molecular Biology* 1, *Bacterial Molecular Networks* (Part 3) 2012;84: 463-479.

4. Chaouiya C, Bérenguier D, Keating SM, Naldi A, van Iersel MP, Rodriguez N, et al. SBML qualitative models: a model representation format and infrastructure to foster interactions between qualitative modelling formalisms and tools. BMC Systems Biology 2013;7:135.

5. Thieffry, D Sánchez L Alternative epigenetic states understood in terms of specific regulatory structures. In: Van Speybroeck L, Van de Vijver G and De Waele D (eds).

*Epigenesis to Epigenetics. The Genome in Context* (eds). Annals of the New York Academy of Sciences, New York, USA, 2002;981: 135-153.

6. Fauré A, Naldi A, Chaouiya C, Thieffy D. Dynamical analysis of a generic Boolean model for the control of the mammalian cell cycle Bioinformatics 2006;22:124–131.

7. Bérenguier D, Chaouiya C, Monteiro PT, Naldi A, Remy E, Thieffry D, et al. Dynamical modeling and analysis of large cellular regulatory networks. Chaos 2013;23: 025114.

8. Moniot B, Declosmenil F, Barrionuevo F, Scherer G, Aritake K, Malki S, et al. The PGD2 pathway, independently of FGF9, amplifies SOX9 activity in Sertoli cells during male sexual differentiation. Development 2009;136: 1813–1821.

9. Barrionuevo F, Georg I, Scherthan H, Lécureuil C, Guillou F, Wegner M, et al. Testis cord differentiation after the sex determination stage is independent of Sox9 but fails in the combined absence of Sox9 and Sox8. Dev Biol 2009;327: 301-312.

10. Chaboissier MC, Kobayash A, Vidal VIP, Lützkendorf S, van de Kant HJG, Wegner M, et al. Functional analysis of Sox8 and Sox9 during sex determination in the mouse. Development 2004;131: 1891-1901.

11. Sock E, Schmidt K, Hermanns-Borgmeyer I, Bösl MR, Wegner M. Idiopathic weight reduction in mice deficient in the high-mobility group transcription factor Sox8. Mol Cell Biol 2001;21: 6951–6959.

12. O'Bryan MK, Takada S, Kennedy CL, Scott G, Harada S, Ray MK, et al. Sox8 is a critical regulator of adult Sertoli cell function and male fertility. Dev Biol 2008;316: 359–370.

13. Matson CK, Murphy MW, Sarver AL, Griswold MD, Bardwell VJ, Zarkower D. DMRT1 prevents female reprogramming in the postnatal mammalian testis. Nature. 2011;476:101–104.

14. Ikeda, Y, Shen WH, Ingraham HA, Parker KL. Developmental Expression of Mouse Steroidogenic Factor-1, an Essential Regulator of the Steroid Hydroxylases. Mol Endocrinol 1994;8: 654-662.

15. Wilhelm D, Englert C. The Wilms tumor suppressor WT1 regulates early gonad development by activation of *Sf1*. Genes Dev 2002;16:1839–1851.

16. Larney C, Bailey TL, Koopman P. Switching on sex: transcriptional regulation of the testis-determining gene Sry. Development. 2014;141:2195–2205.

17. Pilon, N, Daneau I, Paradis V, Hamel F, Lussier JG, Viger RS, et al. Porcine SRY promoter is a target for steroidogenic factor 1. Biol Reprod 2003;68: 1098-1106.

18. Miyamoto Y, Taniguchi H, Hamel F, Silversides DW, Viger RS. A GATA4/WT1 cooperation regulates transcription of genes required for mammalian sex determination and differentiation. BMC Mol Biol 2008;9: 44.

19. Luo X, Ikeda Y, Parker KL. A Cell-Specific Nuclear Receptor Is Essential for Adrenal and Gonadal Development and Sexual Differentiation. Cell 1994;77: 481-490.

20. Sekido R, Bar I, Narváez V, Penny G, Lovell-Badge R. SOX9 is up-regulated by the transient expression of SRY specifically in Sertoli cell precursors. Dev Biol. 2004;274:271–279.

21. Wilhelm, D, Martinson F, Bradford S, Wilson MJ, Combes AN, Beverdam A, et al. Sertoli cell differentiation is induced both cell-autonomously and through prostaglandin signaling during mammalian sex determination. Dev Biol 2005;287: 111-124.

22. Sekido R, Lovell-Badge R. Sex determination involves synergistic action of SRY and SF1 on a specific Sox9 enhancer. Nature. 2008;453:930-934.

23. Vidal VPI, Chaboissier MC, de Rooij DG, Schedl A. Sox9 induces testis development in XX transgenic mice. Nature Genet 2001;28: 216-217.

24. De Santa Barbara, P, Bonneaud N, Boizet B, Desclozeaux M, Moniot B, Sudbeck P, et al. Direct interaction of SRY-related protein SOX9 and steroidogenic factor 1 regulates transcription of the human anti-Müllerian hormone gene. Mol Cell Biol 1998;18: 6653-65.

25. Shen JHC, Ingraham HA. Regulation of the Orphan Nuclear Receptor Steroidogenic Factor 1 by Sox Proteins. Mol Endocrinol 2002;16:529–540.

26. Kim Y, Kobayashi A, Sekido R, DiNapoli L, Brennan J, Chaboissier MC, et al. Fgf9 and Wnt4 act as antagonistic signals to regulate mammalian sex determination. PLoS Biol. 2006;4:e187.

27. Barrionuevo, F, Bagheri-Fam S, Klattig J, Kist R, Taketo MM, Englert C, et al. Homozygous Inactivation of Sox9 Causes Complete XY Sex Reversal in Mice. Biol Reprod 2006;74: 195–201.

28. Kim Y, Bingham N, Sekido R, Parker KL, Lovell-Badge R, Capel B. Fibroblast growth factor receptor 2 regulates proliferation and Sertoli differentiation during male sex determination. Proc Natl Acad Sci USA. 2007;104:16558–16563.

29. Bagheri-Fam S, Sim H, Bernard P, Jayakody I, Taketo MM, Scherer G, et al. Loss of Fgfr2 leads to partial XY sex reversal. Dev Biol. 2008;314:71–83.

30. Jameson SA, Lin YT, Capel B. Testis development requires the repression of Wnt4 by Fgf signaling. Dev Biol 2012;370: 24-32.

31. Tevosian SG, Manuylov NL. To ß or Not to ß: Canonical ß-Catenin Signaling Pathway and Ovarian Development. Dev Dyn. 2008;237:3672–3680.

32. Akiyama H, Lyons JP, Mori-Akiyama Y, Yang X, Zhang R, Zhang Z, et al. Interactions between Sox9 and ß-catenin control chondrocyte differentiation. Genes Dev 2004;18: 1072-1087.

33. Ottolenghi C, Pelosi E, Tran J, Colombino M, Douglass E, Nedorezov T, et al. Foxl2 is required for commitment to ovary differentiation. Human Mol Genet. 2005;14:2053–2062.

34. Uhlenhaut NH, Jakob S, Anlag K, Eisenberger T, Sekido R, Kress J, et al. Somatic sex reprogramming of adult ovaries to testes by FOXL2 ablation. Cell. 2009;139: 1130–1142.

35. Lei N, Karpova T, Hornbaker KI, Rice DA, Heckert LL. Distinct Transcriptional Mechanisms Direct Expression of the Rat Dmrt1 Promoter in Sertoli Cells and Germ Cells of Transgenic Mice. Biol Reprod 2009;81: 118–125.

36. Raymond C S, Murphy MW, O'Sullivan MG, Bardwell VJ, Zarkower D. *Dmrt1*, a gene related to worm and fly sexual regulators, is required for mammalian testis differentiation. Genes Dev. 2000;14:2587–2595.

37. Lavery R, Lardenois A, Ranc-Jianmotamedi F, Pauper E, Gregoire EP, Vigier C, et al. XY Sox9 embryonic loss-of-function mouse mutants show complete sex reversal and produce partially fertile XY oocytes. Dev Biol 2011;354: 111–122.

38 Schmidt D, Ovitt CE, Anlag K, Fehsenfeld S, Gredsted L, Treier AC, et al. The murine winged-helix transcription factor Foxl2 is required for granulosa cell differentiation and ovary maintenance. Development. 2004;131:933–942.

39. Herpin A, Schartl M. Sex Determination: Switch and Suppress. Current Biology 2011;21: R656-R659.

40. Viger RS, Mertineit C, Trasler JM, Nemer M. Transcription factor GATA-4 is expressed in a sexually dimorphic pattern during mouse gonadal development and is a potent activator of the Mullerian inhibiting substance promoter. Development 1998;125: 2665–2675.

41. Lei N, Heckert LL. Gata4 Regulates Testis Expression of Dmrt1. Mol Cell Biol 2004;24: 377-388.

42. Manuylov NL, Zhou B, Ma Q, Fox SC, Pu WT, Tevosian SG. Conditional ablation of Gata4 and Fog2 genes in mice reveals their distinct roles in mammalian sexual differentiation. Dev Biol 2011;353: 229–241.

43. Kim S, Bardwell VJ, Zarkower D. Cell type-autonomous and non-autonomous requirements for Dmrt1 in postnatal testis differentiation. Dev Biol. 2007;307:314–327.

44. Luo X, Ikeda Y, Parker KL. A Cell-Specific Nuclear Receptor Is Essential for Adrenal and Gonadal Development and Sexual Differentiation. Cell 1994;77: 481-490.

45. Gubbay J, Vivian N, Economou A, Jackson D, Goodfellow P, Lovell Badge R. Inverted repeat structure of the Sry locus in mice. Proc. Natl. Acad. Sci. USA 1992;89: 7953–7957.

46. Koopman P, Gubbay J, Vivian N, Goodfellow P, Lovell-Badge R. Male development of chromosomally female mice transgenic for *Sry*. Nature. 1991;351:117–121.

47. Vidal VPI, Chaboissier MC, de Rooij DG, Schedl A. Sox9 induces testis development in XX transgenic mice. Nature Genet 2001;28: 216-217.

48. Schmahl J, Kim Y, Colvin JS, Ornitz DM, Capel B. Fgf9 induces proliferation and nuclear localization of FGFR2 in Sertoli precursors during male sex determination. Development. 2004;131:3627–3636.

49. Colvin JS, Green RP, Schmah J, Capel B, Ornitz DM. Male-to-Female Sex Reversal in Mice Lacking Fibroblast Growth Factor 9. Cell 2001;104: 875-889.

50. Vainio S, Heikkila M, Kispert A, Chin N, McMahon AP. Female development in mammals is regulated by Wnt-4 signalling. Nature. 1999;397:405–409.

51. Jordan BK, Shen JHC, Olaso R, Ingraham HA, Vilain E. Wnt4 overexpression disrupts normal testicular vasculature and inhibits testosterone synthesis by repressing steroidogenic factor 1/ß-catenin synergy. Proc Natl Acad Sci USA. 2003;100:10866–10871.

52. Maatouk, DM, DiNapoli L, Alvers A, Parker KL, Taketo MT, Capel B. Stabilization of ß-catenin in XY gonads causes male-to-female sex-reversal. Human Mol Genet 2008;17:2949–2955.

53 Lindeman RE, Gearhart MD, Minkina A, Krentz AD, Bardwell VJ, Zarkower D. Sexual Cell-Fate Reprogramming in the Ovary by DMRT1. Current Biology 2015;25: 764–771.

54. Nicol B, Yao HHC. Gonadal Identity in the Absence of pro-Testis Factor SOX9 and pro-Ovary Factor beta-catenin in mice. Biol Rreprod. 2015;93:1-12.

55. Lavery R, Chassot AA, Pauper E, Gregoire EP, Klopfenstein M, de Rooij DG, et al. Testicular differentiation occurs in absence of Rspondin1 and Sox9 in mouse sex reversals. PLoS Genet 2012;8:e1003170.

56. Schepers GE, Bullejos M, Hosking BM, Koopman P. Cloning and characterisation of the *Sry*-related transcription factor gene *Sox8*. Nucleic Acids Res 2000;28: 1473-1480.

57. Schepers GE, Wilson M, Wilhelm D, Koopman P. SOX8 Is Expressed during Testis Differentiation in Mice and Synergizes with SF1 to Activate the *Amh* Promoter *in Vitro.* J Biol Chem 2003;278: 28101-28108.

58. Chaboissier MC, Kobayashi A, Vidal VIP, Lützkendorf S, van de Kant HJKG, Wegner M, et al. Functional analysis of Sox8 and Sox9 during sex determination in the mouse. Development 2004;131:1891-1901.

59. Li Y, Zheng M, Lau YF. The sex-determining factors SRY and SOX9 regulate similar target genes and promote testis cord formation during testicular differentiation. Cell Rep 2014; 8:723-733.

## Legends to Supporting Figures and Tables

Embedded in the supporting figures and tables

**Figure S1.** The male and female genes that have been identified and their proposed interactions involved in the sexual development of the gonad in placental mammals. Normal green and blunt red arrows represent positive and negative interactions, respectively. Grey nodes were subjected to simplification in the model of Figure 1 (see main text).

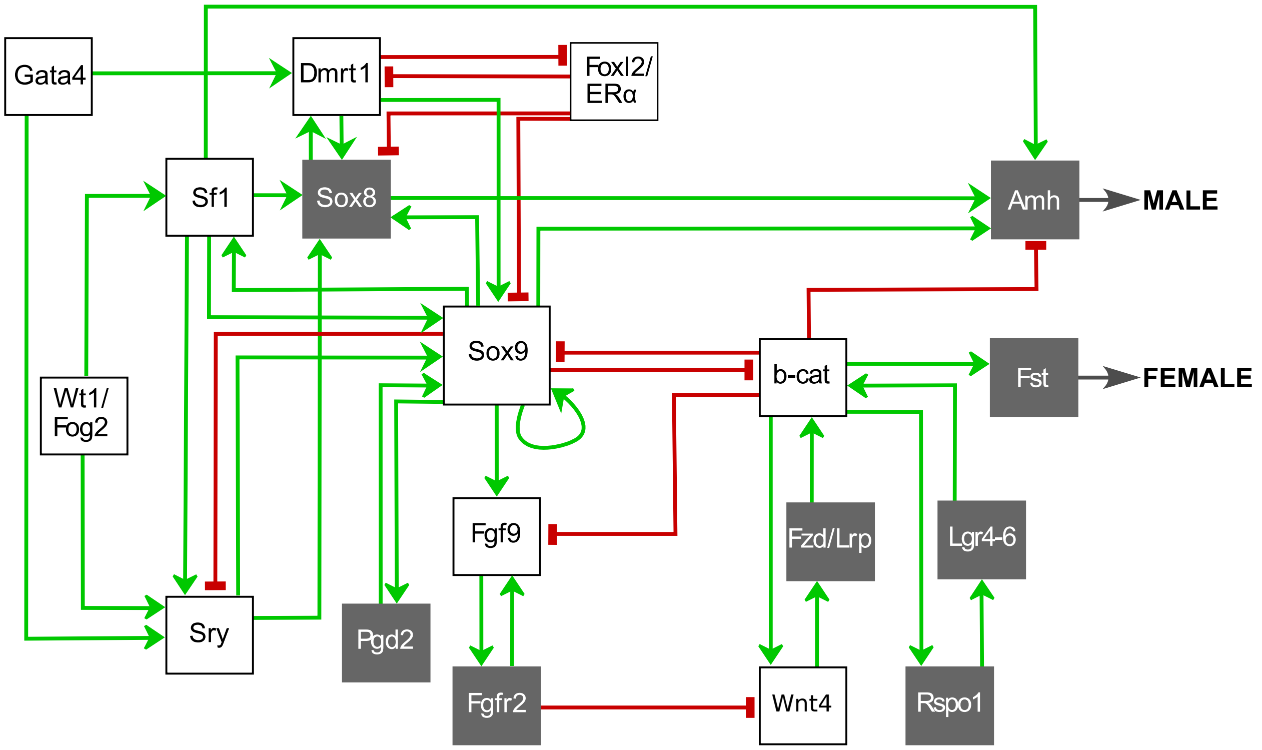

**Figure S2.** Hierarchical transition graphs (see Supplemtary Text) revealing bifurcations in the discrete dynamics and the required restrictions on the delays to ensure the reachability of the expected final stable state (see main text). The final stable states are given as vector states, with the values of the 14 model components, in the following order: Y, WT1, AS, AS, Gata4, AF, IW, Sf1, Sox9, Fgf9, Wnt4,ß-cat,Dmrt1, Foxl2. The signs + and - associated to the gene names indicate updates increasing and decreasing the corresponding gene levels.

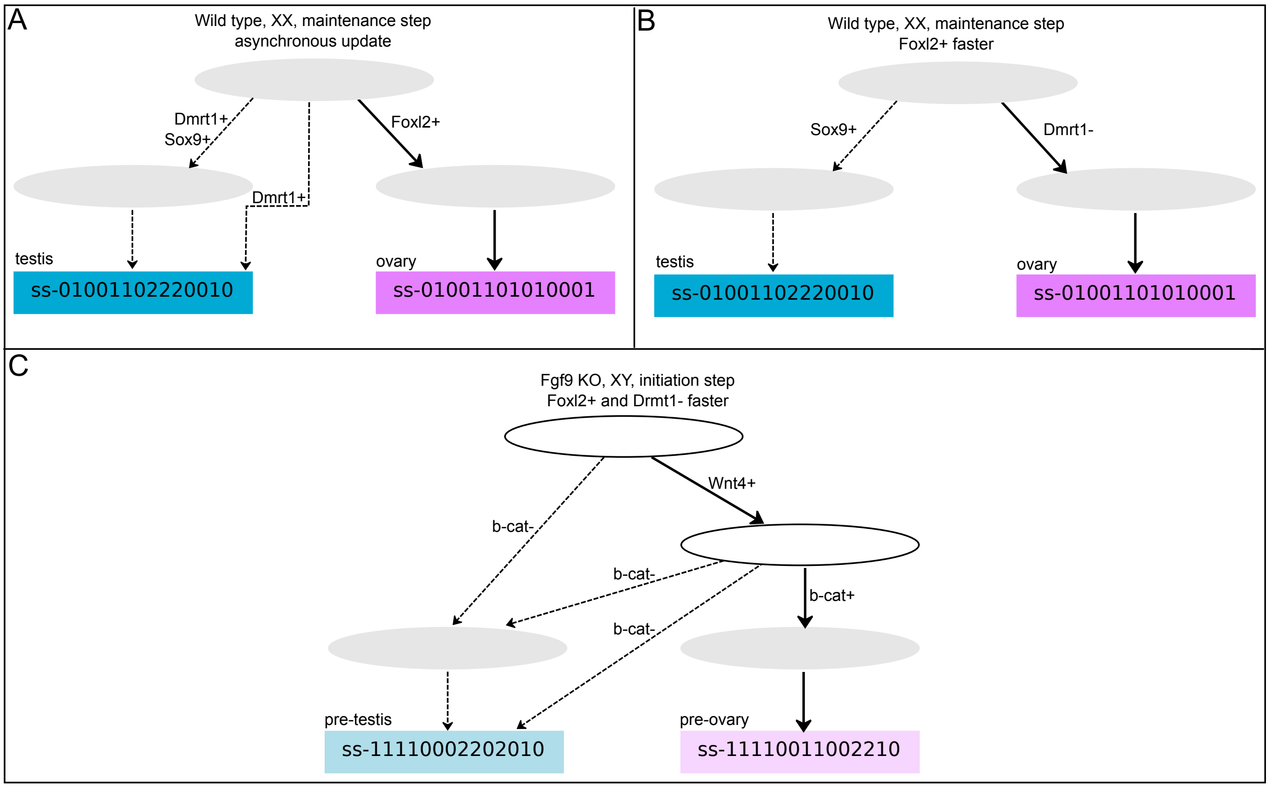

**Figure S3.** Final states reached by the gene network and their corresponding phenotypes (testis, ovary) for the gonad under mutant conditions (loss--of-function and gain-of-function) of the developmental signals. Phenotypes resulting from mutations of the target genes of these signals are described in Figure 3 (see main text).

| Genotype | *Sf1* | *Sox9* | *Fgf9* | *Wnt4* | *ß-cat* | *Dmrt1* | *Foxl2* | Predicted gonad |
|---|---|---|---|---|---|---|---|---|
| WT XY | 2 | 2 | 2 | 0 | 0 | 1 | 0 | testis |
| WT XX | 1 | 0 | 1 | 0 | 0 | 0 | 1 | ovary |
| AS-KO XY | 1 | 0 | 0 | 1 | 1 | 0 | 1 | ovary |
| AS-GF XX | 1 | 0 | 1 | 0 | 0 | 0 | 1 | ovary |
| GATA4-KO XY | 1 | 0 | 1 | 0 | 0 | 0 | 1 | ovary |
| GATA4-GF XX | 2 | 2 | 2 | 0 | 0 | 1 | 0 | testis |
| IW-KO XX | 1 | 0 | 1 | 1 | 1 | 0 | 1 | ovary |
| IW-GF XY | 2 | 2 | 2 | 0 | 0 | 1 | 0 | testis |
| IW-GF XX | 2 | 2 | 2 | 0 | 0 | 1 | 0 | testis |
| AF-KO XX | 2 | 2 | 2 | 0 | 0 | 1 | 0 | testis |
| AF-GF XY | 2 | 2 | 2 | 0 | 0 | 1 | 0 | testis |

0
1 (when this is not the max value)
max value

**Table S1.** Symbols and names of the genes

| **Symbol** | **Name of the gene** |
|---|---|
| Gata4 | GATA binding protein 4 |
| Wt1 | Wilms tumor 1 |
| Fog2 | Zinc Finger Protein, FOG Family Member 2 |
| Sf1 | Steroidogenic factor 1 |
| Sry | Sex-determining region Y |
| Sox8 | Sry-box 8 |
| Sox9 | Sry-box 9 |
| Pgd2 | Prostaglandin D2 synthase |
| Fgf9 | Fibroblast growth factor 9 |
| Fgfr2 | Fibroblast growth factor receptor 2 |
| Wnt4 | Wingless related MMTV integration site 4 |
| Fzd | G protein-coupled receptors, Class F frizzled |
| Lrp | Low density lipoprotein receptor-related protein |
| Rspo1 | R-sponding 1 |
| Lgr4-6 | Leucine-rich repeat-containing G-protein coupled receptor 4-6 |
| b-cat | Catenin beta-1 |
| Dmrt1 | Doublesex- and mab-3-related transcription factor 1 |
| Foxl2 | Forkhead-domain transcription factor L2 |
| ERα | oestrogen receptor alfa |
| Amh | Anti-Müllerian hormone |
| Fst | Follistatin |

**Table S2.** Maximal values and logical parameters defining the effect of regulatory interactions, for each model component. Interactions are denoted by the name of their source node, together with the level(s) for which they are operative. The value of a parameter defines the effect of a given combination of interactions (set in parentheses) operating on a given component. For example, $K_{Dmrt}$({Gata4,1},{Sox9,2})=1 indicates that the target value of Dmrt1 is 1, in the presence of Gata4 and Sox9 (both at level 1) and in the absence of Sf1. Note that only the non-zero parameters are listed. The lower part of the table relates to components of the 2-cell network.

| Network element | Maximal value | Logical parameter values |
|---|---|---|
| Y | 1 | constant to initial value |
| Wt1 | 1 | constant to initial value |
| AS | 1 | constant to initial value |
| Gata4 | 1 | constant to initial value |
| AF | 1 | constant to initial value |
| IW | 1 | constant to initial value |
| Sry | 1 | $K_{Sry}$({Y,1},{Wt1,1},{AS,1},{Sf1,1},{Gata4,1})=1 |
| Sf1 | 2 | $K_{Sf1}$({Wt1,1})=1<br>$K_{Sf1}$({Wt1,1},{Sox9,2})=2 |
| Sox9 | 2 | $K_{Sox9}$({Sf1,1})=1<br>$K_{Sox9}$({Sf1,1}, {b-cat,1})=1<br>$K_{Sox9}$({Sf1,1}, {b-cat,1},{Sox9,1})=1<br>$K_{Sox9}$({Sf1,1},{Sry,1})=2<br>$K_{Sox9}$({Sf1,1},{b-cat,1},{Sry,1})=2<br>$K_{Sox9}$({Sf1,1},{Sry,1},{Sox9,1})=2<br>$K_{Sox9}$({Sf1,1},{b-cat,1},{Sry,1},{Sox9,1})=2<br>$K_{Sox9}$({Sf1,1},{Sox9,1})=2 |
| Fgf9 | 2 | $K_{Fgf9}$({Fgf9,1})=1<br>$K_{Fgf9}$({Sox9,2})=1<br>$K_{Fgf9}$({Fgf9,1},({Sox9,2})=2 |
| Wnt4 | 2 | $K_{Wnt4}$({Wnt4,1},{Fgf9,1})=1<br>$K_{Wnt4}$({Wnt4,1})=2 |
| b-cat | 2 | $K_{b\text{-}cat}$({Wnt4,1})=1<br>$K_{b\text{-}cat}$({Wnt4,2})=2 |
| Dmrt1 | 1 | $K_{Dmrt}$({Gata4,1})=1<br>$K_{Dmrt}$({Gata4,1},{Sf1,1})=1<br>$K_{Dmrt}$({Gata4,1},{Sox9,2})=1<br>$K_{Dmrt}$({Gata4,1},{Sox9,2},{Sf1,1})=1<br>$K_{Dmrt}$({Sox9,2},{Sf1,1})=1 |
| Foxl2 | 1 | $K_{Dmrt}$({AF,1})=1 |
| Fgf9_c | 2 | $K_{Fgf9\_c}$({Sox9_c,2})=1<br>$K_{Fgf9\_c}$({Fgf9r_c,1})=1<br>$K_{Fgf9\_c}$({Sox9_c,2},{Fgf9r_c,1})=2 |
| Fgf9r_c | 2 | $K_{Fgf9r\_c}$({Fgf9_c,1})=1<br>$K_{Fgf9r\_c}$({Fgf9_c,1},({Fgf9_p,2})=2 |
| Wnt4_c | 2 | $K_{Wnt4\_c}$({Wnt4_c,1},{Fgf9r_c,1})=1<br>$K_{Wnt4\_c}$({Wnt4_c,1})=2 |
| Fgf9_p | 2 | $K_{Fgf9\_p}$({Sox9_p,2})=1<br>$K_{Fgf9\_p}$({Fgf9r_p,1})=1<br>$K_{Fgf9\_p}$({Sox9_p,2},{Fgf9r_p,1})=2 |
| Fgf9r_p | 2 | $K_{Fgf9r\_p}$({Fgf9_p,1})=1<br>$K_{Fgf9r\_p}$({Fgf9_p,1},({Fgf9_c,2})=2 |
| Wnt4_p | 2 | 2$K_{Wnt4_p}$({Wnt4_p,1},{Fgf9r_p,1})=1<br>$K_{Wnt4\_p}$({Wnt4_p,1})=2 |

| | Y | Wt1 | AS | G4 | AF | IW | Sry | Sf1 | Sox9 | Fgf9 | Wnt4 | b-cat | Dmrt1 | Foxl2 | Probability |
|---|---|---|---|---|---|---|---|---|---|---|---|---|---|---|---|
| Male Init | 1 | 1 | 1 | 1 | 0 | 0 | 0 | 2 | 2 | 2 | 1 | 0 | 1 | 0 | **0.5344** |
| | | | | | | | 0 | 2 | 2 | 2 | 0 | 0 | 1 | 0 | 0.3348 |
| | | | | | | | 1 | 1 | 0 | 0 | 2 | 2 | 1 | 0 | 0.1308 |
| Male Maint | 1 | 1 | 0 | 0 | 1 | 1 | 0 | 1 | 0 | 1 | 0 | 0 | 0 | 1 | 0.5926 |
| | | | | | | | 0 | 1 | 0 | 0 | 0 | 0 | 0 | 1 | 0.2362 |
| | | | | | | | 0 | 2 | 2 | 2 | 0 | 0 | 1 | 0 | **0.1712** |
| Female Init | 0 | 1 | 1 | 1 | 0 | 0 | 0 | 1 | 1 | 1 | 1 | 1 | 1 | 0 | **0.3474** |
| | | | | | | | 0 | 2 | 2 | 2 | 1 | 0 | 1 | 0 | 0.3352 |
| | | | | | | | 0 | 1 | 0 | 0 | 2 | 2 | 1 | 0 | 0.1628 |
| | | | | | | | 0 | 2 | 2 | 2 | 1 | 0 | 1 | 0 | 0.1546 |
| Male Maint | 0 | 1 | 0 | 0 | 1 | 1 | 0 | 1 | 0 | 1 | 0 | 0 | 0 | 1 | **0.5936** |
| | | | | | | | 0 | 1 | 0 | 0 | 0 | 0 | 0 | 1 | 0.2344 |
| | | | | | | | 0 | 2 | 2 | 2 | 0 | 0 | 1 | 0 | 0.172 |

**Table S3:** Model stable states complying with selected combinations of the temporal signals (left column). Color code indicates the male (blue) *versus* female (pink) situation (with the presence/absence of Y), light colors correspond to the initiation phase, whereas darker colors correspond to the maintenance phase. For each such combination, there are 3 to 4 potential stable states, the one reached in our simulations (i.e., selecting an initial condition) is highlighted with the appropriate color. The latest column indicates the probability of reaching the corresponding stable state when sampling the initial condition over the whole state space (using an asynchronous update). Remarkably, the Sertoli phenotype has a low probability (0.1712), suggesting the importance of the initiation phase and the selection of the appropriate initial condition.
